# A population-representative serosurvey estimating vaccine-induced immunity against measles, rubella, hepatitis B and severe acute respiratory syndrome coronavirus 2 in Timor-Leste

**DOI:** 10.1016/j.lansea.2024.100525

**Published:** 2025-02-25

**Authors:** Paul Arkell, Maria Y. Tanesi, Nelson Martins, Nelia Gomes, Tessa Oakley, Vanessa Solano, Michael David, Salvador Amaral, Sarah L. Sheridan, Teem-Wing Yip, Anthony D.K. Draper, Nevio Sarmento, Endang Soares da Silva, Lucsendar Alves, Suellen Nicholson, Vicki Stambos, Kate Pedrina, Carlito Freitas, Filipe de Neri Machado, Celia A. Gusmão, Ismael da Costa Barreto, Nicholas S.S. Fancourt, Kristine Macartney, Jennifer Yan, Joshua R. Francis

**Affiliations:** a Global and Tropical Health Division, Menzies School of Health Research, Charles Darwin University, Dili, Timor-Leste; b Research Institute for the Environment and Livelihoods, Charles Darwin University, Darwin, Australia; c Daffodil Centre, The University of Sydney, a Joint Venture with Cancer Council New South Wales, Sydney, NSW, Australia; d School of Medicine & Dentistry, Griffith University, Gold Coast, QLD, Australia; e National Centre for Immunisation Research and Surveillance (NCIRS), Westmead, NSW, Australia; f College of Medicine and Public Health, Flinders University, Australia; g Northern Territory Centre for Disease Control, Darwin, Australia; h National Centre for Epidemiology and Population Health, Australian National University, Canberra, Australia; i Laboratório Nacional da Saúde, Dili, Timor-Leste; j Victorian Infectious Diseases Reference Laboratory, Royal Melbourne Hospital, Peter Doherty Institute for Infection and Immunity, Melbourne, Australia; k Ministry of Health, Dili, Timor-Leste; l Hospital Nacional Guido Valadares, Dili, Timor-Leste; m Faculty of Medicine and Health, University of Sydney, Australia

**Keywords:** Vaccine preventable diseases, Serosurveillance, Immunisation, Global health

## Abstract

**Background:**

Serosurveillance can be used to assess population immunity to vaccine-preventable diseases (VPDs). This study aimed to determine seroprevalence of four VPDs across Timor-Leste and identify immunity gaps.

**Methods:**

A population-representative three-stage cluster random sample of census-enumerated households were visited between October 2021 and February 2023. Occupants aged above one year were tested for measles immunoglobulin G (IgG), rubella IgG, hepatitis B surface antibody (HBsAb), hepatitis B core antibody (HBcAb) and severe acute respiratory syndrome 2 (SARS-CoV-2) IgG, using serological assays with *a priori* determined cut-offs. Sample- and response-weighted mixed effects logistic regression models were used to estimate seroprevalence in relevant age-strata.

**Findings:**

Of 2613 eligible households, 1908 (73.0%) participated. Of 8427 occupants, 4750 (56.4%) participated. Measles IgG seroprevalence was low among children, particularly those aged 10–14 (33.2%, 95% confidence interval (CI) 27.8–38.6%). Rubella IgG seroprevalence was high in all ages (93.2%, 95% CI 92.2–94.2%). SARS-CoV-2 IgG seroprevalence was high, including in young children not eligible for vaccination (74.0%, 95% CI 70.4–77.6%). HBsAb seroprevalence was lowest among adolescents aged 15–19 (12.1%, 95% CI 6.8–17.5%) but higher among younger children, who also had low HBcAb seroprevalence.

**Interpretation:**

The pattern of measles immunity is consistent with low virus transmission and suboptimal childhood vaccine uptake. These data have informed supplementary immunisation activities. High rubella IgG seroprevalence suggests recent or ongoing virus transmission and a need for congenital rubella syndrome surveillance. Hepatitis B data provide evidence of recent improvements in vaccine-induced immunity and protection. This study demonstrates how serosurveillance can directly influence national vaccine strategies.

**Funding:**

This study was funded by the Department for Foreign Affairs and Trade, Australian Government (Complex Grant Agreement Number 75889).

**Research in context**

**Evidence before this study**

Serological surveys can be used to inform understanding of population-level immunity against vaccine-preventable diseases (VPDs) resulting from vaccination or previous infection. Nationally representative serosurveys have been conducted in several low-middle income countries; a 2016 review by Dimech and Mulders identified 13 publications including measles serosurveys from “least developed countries”. Sampling frames vary considerably across such studies, and the lack of standardisation makes direct comparisons difficult. However, in settings where nationally representative serosurveys have been conducted, there is evidence that results from these may be used to inform vaccination policy decisions that are responsive to local epidemiology.

**Added value of this study**

This study provides population-representative data on immunity to four priority VPDs (measles, rubella, hepatitis B, severe acute respiratory virus 2) in Timor-Leste. It identified significant immunity gaps for measles and hepatitis B, which were not apparent from vaccine coverage estimates, and evidence that suggests ongoing rubella transmission, despite recent declaration of rubella elimination.

**Implications of all the available evidence**

Identification of a measles immunity gap has led to implementation of a supplementary immunisation activity. Efforts to improve uptake of routine childhood vaccination are essential, and given identified immunity gaps, an ongoing focus on surveillance for VPDs must be maintained in Timor-Leste.

## Introduction

Serological testing can determine whether individuals have circulating antibody (humoral immunity) to vaccine-preventable diseases (VPDs) including measles, rubella, severe acute respiratory syndrome coronavirus 2 (SARS-CoV-2) and hepatitis B. In some cases, a specific level has been associated with protection from infection (correlate-of-protection).^1^^,^^2^ Community-based serological surveillance (serosurveillance) can augment understanding of population-level immunity achieved through vaccination or derived from prior infection. This may indicate the effectiveness of vaccine programmes and/or identify sub-groups more susceptible to infection.^3^^,^^4^ The results of serosurveys can be used to guide supplementary immunisation activities (SIAs), tailor routine immunisation service delivery or plan targeted catch up campaigns.^5^

The Democratic Republic of Timor-Leste (Timor-Leste) is a half-island nation between Australia and Indonesia with a population of 1.3 million people. The Expanded Program on Immunisation (EPI) was introduced before 1989, when Timor-Leste was an Indonesian province. Following this, periods of political instability led to disruption in healthcare infrastructure, including the near cessation of routine vaccine delivery around the time of regained independence in 2002. Thereafter, EPI was reinstated as part of a national vaccination programme, initially with single-dose measles vaccination. Hepatitis B vaccination in infancy (three doses) was introduced by 2007 and birth dose hepatitis B vaccine was introduced in 2016, along with combined measles-rubella (MR) vaccination (two doses at ages 9 and 18 months). In July 2018, an SIA offered children aged between 9 and 59 months a single MR vaccine dose. Vaccination against SARS-CoV-2 began in adults in April 2021 and was offered to adolescents aged 12 years and older from October 2021. All vaccines currently in use in Timor-Leste are WHO-pre qualified and sourced through the United Nations Children's Fund (UNICEF). The authors of this manuscript (many of whom are key stakeholders in the Timorese vaccination programme in Timor-Leste) understand that this is also true of previously used vaccines, at least since independence in 2002.

In Timor-Leste, public healthcare services are free for Timorese nationals. This includes routine vaccinations, with children being required to travel to community health centres (CHCs) to receive their vaccinations. Families do not receive compensation for travel, time off work, or other related expenses. The country's geographical conditions, a lack of transport infrastructure, community health worker shortages, and various social and behavioural drivers may also affect vaccine accessibility and uptake.

Data relating to vaccine uptake in Timor-Leste are limited due to data quality issues and lack of accurate denominator data, justifying vaccination coverage surveys. In 2018 and 2023, vaccination coverage surveys found variable uptake of MR and hepatitis B vaccines across different municipalities and large discrepancies between ‘valid’ (confirmed by review of vaccination card) and ‘crude’ (based on maternal history alone) doses. These studies were not accompanied by serological testing.^6^^,^^7^ In 2021, a study of healthcare workers found lower than expected measles and hepatitis B surface antibody (HBsAb) seroprevalence, but high SARS-CoV-2 seroprevalence.^8^^,^^9^ A survey of residual serum samples from laboratories across Timor-Leste during 2021 indicated that SARS-CoV-2 seroprevalence had reached high levels. However, in this study samples were collected opportunistically after being taken for other clinical reasons, therefore the study lacked a systematic sampling design.^10^ Timor-Leste was verified by the World Health Organization (WHO) as having eliminated measles in 2018 but remains vulnerable to re-introduction if optimal population immunity is not reached and maintained.^11^

The primary aim of this study was to determine measles, rubella, SARS-CoV-2, and hepatitis B virus seroprevalence in relevant age-strata of the population in Timor-Leste. The secondary aim of this study was to explore individual and household determinants of seropositivity. These were chosen based on discussion with local research and public health authorities, within the context of Timor-Leste's national vaccination programme.

## Methods

Detailed study methods have been published previously.^12^

### Sampling strategy

This study used the 2015 Timor-Leste national population census as its sampling frame. One-hundred-and-thirteen enumeration areas (EAs, roughly equivalent to villages) were randomly selected from all EAs in the country (N = 2320). These were selected from all municipalities in Timor-Leste (N = 13) with probability proportional to municipality population. The number of targeted EAs changed between first submission of the study protocol for publication (when N = 112) and fieldwork starting (when N = 113). This was because the number to be selected from Manatuto municipality (which had the smallest population) was rounded up (to 4 EAs), rather than down (to 3 EAs), giving slightly better representation in Manatuto and ensuring at least four EAs in each municipality were to be visited. Up to twenty-three households were selected from each EA by simple random selection. Occupants aged above one year were eligible to participate. This strategy was designed to fulfil a target sample size such that enough individuals from each of five key age strata (1–4, 5–14, 15–24, 25–40, and 41+ years) would be included. The calculation was based upon an expected seroprevalence of 50%, a required precision of ±6% for the 95% confidence interval (CI), and an overall design effect of 4. It provided a target sample size for each stratum of 1120 individuals (total sample size 5600 individuals).

### Data and sample collection

Fieldwork occurred between October 2021 and February 2023. Municipalities were visited sequentially and targeted households were located using global positioning system (GPS)-connected electronic tablets and bespoke printed maps. Households were visited three times or until all eligible occupants had either participated or refused participation.

First, ‘household level data’ were collected from the head-of-household including age and gender of all occupants. Second, ‘individual level data’ were collected from each participating occupant using a structured interview questionnaire with responses entered into a REDCap database.^13^

Serum samples were collected by phlebotomy, centrifuged, and refrigerated at 2–8 °C until analysis at Laboratório Nacional da Saúde (LNS) in Dili, Timor-Leste.

### Sample analysis

Samples were analysed for antibody targets relevant to measles, rubella, hepatitis B and SARS-CoV-2. Measles IgG was detected using Euroimmun® Anti-Measles Virus ELISA (IgG) assay. This assay had undergone validation by the manufacturer with WHO-recognised standard material. A cut-off of >120 IU/L was chosen because this is the most commonly cited correlate-of-protection.^2^^,^^14^, ^15^, ^16^ A subset of 36 samples were sent to the WHO Measles Regional Reference Laboratory at The Victorian Infectious Diseases Reference Laboratory (VIDRL), Melbourne, Australia and re-tested, to check concordance of antibody concentrations.

Rubella IgG, SARS-CoV-2 anti-spike IgG, hepatitis B core antibody (HBcAb) and HBsAb were detected using Ortho Clinic Diagnostics® chemiluminescent (IgG) assays on the Vitros EciQ® platform. The manufacturer's recommended cut-offs were used, including >10 IU/mL for each of rubella IgG and HBsAb, which are WHO-recommended correlates-of-protection for these pathogens.^2^^,^^17^, ^18^, ^19^, ^20^

### Data analysis: describing participation

Households were defined as ‘participating households’ if they were successfully visited and one or more occupants agreed to participate. Characteristics of participating households were compared to those of non-participating households. Individuals from whom a serum sample was collected and analysed were included in data analysis as ‘participants’. Characteristics of participants were compared to those of non-participating household occupants.

### Data analysis: defining age strata and vaccine-eligible populations

VPD seroprevalence was estimated for five ‘*a priori-*determined age-strata’ (1–4, 5–14, 15–24, 25–40, and 41+ years) as well as ‘age-strata at 5-year intervals’. With the aim of specifically evaluating immunity from vaccines, we also estimated seroprevalence among the group of individuals who were ‘vaccine-eligible’ for each VPD, based on their age. For measles, rubella, SARS-CoV-2 and hepatitis B, participants aged 1–34, 1–9, 15+, and 1–14 years, respectively, were considered vaccine-eligible. These age boundaries were set based on historical records relating to dates of vaccine introduction into the national programme. They were rounded up so that they aligned with reported age groups in the 2015 national census.

### Data analysis: estimating national and municipal VPD seroprevalence

First, ‘crude positive seroprevalence rates’ (CPRs) were calculated by dividing the number of seropositive participants by the total number of participants within each group. Second, to account for sampling and clustering design effects, ‘model-predicted seroprevalence rates’ (MPRs) were calculated. This was achieved by calculating sampling weights at each level of the sample design (EAs within municipalities, households within EAs and individuals within households), which were the product of probability weights and non-response weights at each level. Then, an overall sampling weight for each participant was calculated as the product of their sampling weights at each level. Following this, a sample-weighted multivariable mixed-effects logistic regression model was fitted for each VPD. In each case, ‘seropositivity’ was the binary dependent variable. ‘Participant age strata’ (5-year interval), ‘participant gender’ and ‘municipality’ were fitted as independent variables with fixed effect, while ‘household number’ and ‘EA number’ were fitted as independent variables with random effect (random intercept, fixed slope). MPRs and 95% confidence intervals (Cis) were computed for each group.

### Data analysis: assessing for potential determinants of VPD seropositivity

Various individual (‘age stratum’, ‘gender’, ‘previous COVID-19 vaccine’, ‘days since SARS-CoV-2 outbreak started in Timor-Leste’), household (‘household occupancy’, ‘log (distance to CHC)’, ‘urban vs. rural location’) and population (‘log (EA population density)’, ‘municipality’) variables were assessed as potential determinants of VPD seropositivity. These were chosen based on their observed association in previous studies. Continuous variables observed to have right-skewed distribution (‘distance to CHC’ and ‘EA population density’) were log-transformed. Under the assumption that factors determining VPD seropositivity are different among vaccine-eligible (compared with vaccine-ineligible) participants, and with the primary aim of this study being to evaluate vaccine-induced immunity, this analysis was undertaken using data from vaccine-eligible participants only. Variables were added to multivariable mixed-effects logistic regression models for each VPD, which also included ‘household’ and ‘EA’ as random effects. Potential multicollinearity was assessed for all models by calculating the variance inflation factor for each independent variable. Additionally, where event sizes were low and confidence intervals were consequently wide for some variables, a sensitivity analysis was undertaken, which compared point and precision estimates generated by modelling with and without these variables.

### Ethical considerations

Participants were given verbal and written information about the purpose of the proposed study and its procedures and provided informed written consent. This study received ethical approval from the Instituto Nacional da Saúde (INS) Research Ethics Committee, Timor-Leste (Reference: 875 MS-INS/DGE/IX/2021) and the Northern Territory Human Research Ethics Committee, Australia (Reference: 2021-4064).

### Role of the funding source

This study was funded by the Department for Foreign Affairs and Trade, Australian Government (Complex Grant Agreement Number 75889), which had no role in study design; in the collection, analysis, and interpretation of data; in the writing of the report; or in the decision to submit the paper for publication.

## Results

### Recruitment

**Fig. 1:**
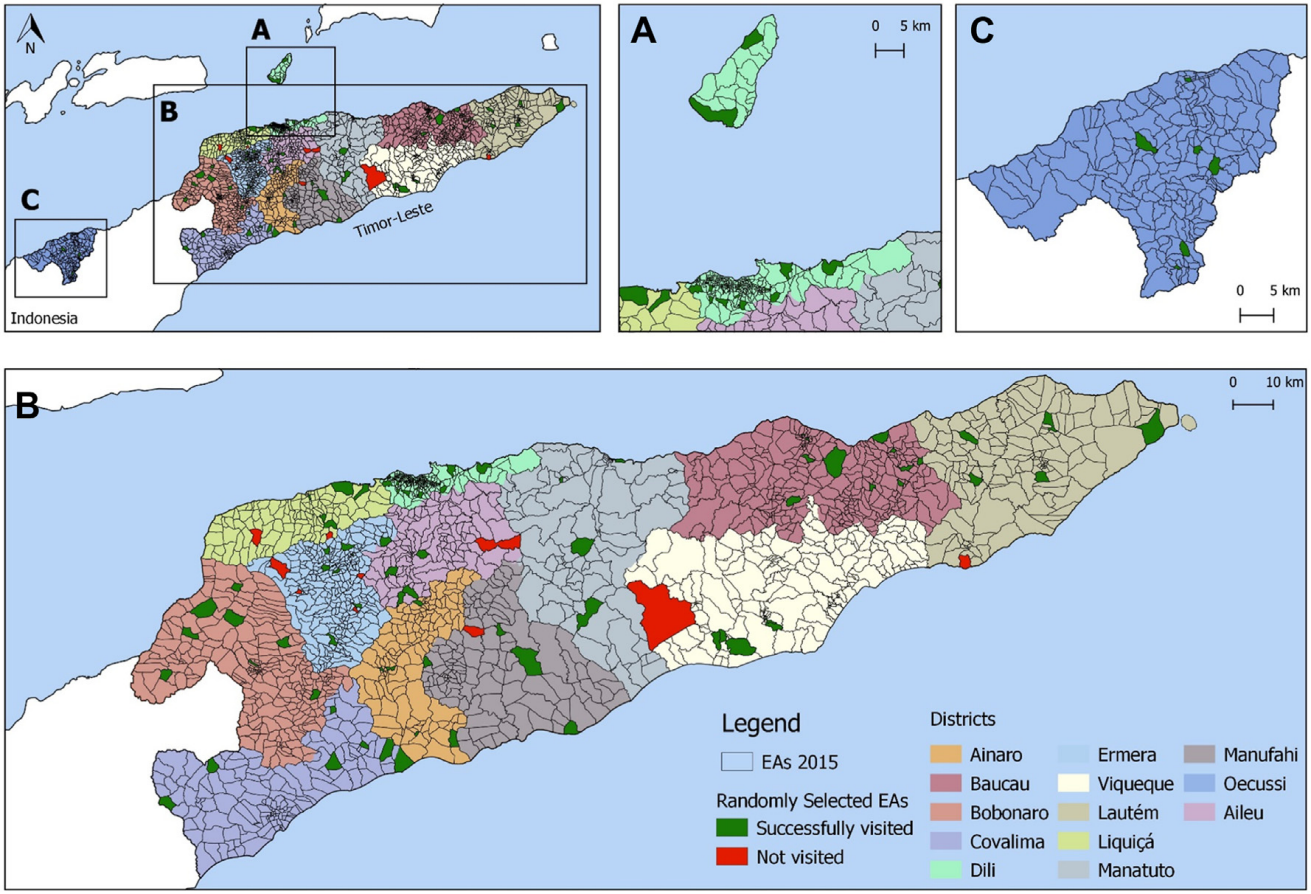
Maps of Timor-Leste showing selected enumeration areas (EAs) within municipalities. B shows the main land area of Timor-Leste, i.e. the Eastern part of Timor Island. A shows Dili municipality, which is on the northern coast and is Timor-Leste's most populous area. At the time of the study Dili municipality also included the island of Atauro. C shows the Special Administrative Region of Oecussi, which is separated from the rest of Timor-Leste by Indonesian West Timor.

One-hundred-and-two (90.3%) EAs were successfully visited. Eight (7.1%) were not visited because of ‘poor weather and road damage’, two (1.8%) because of ‘inadequate time’, and one (0.9%) because of ‘field staff illness’. Fig. 1 shows the locations of selected EAs across Timor-Leste.

Of the 2558 targeted households, 1853 (72.4%) participated. Sixty (3.2%) of these were adjacent ‘replacement houses’, chosen because the original building was derelict, destroyed or no longer a dwelling. Fifty-five additional households were recruited because more than one mutually exclusive group of individuals were living in separate parts of the same building. Therefore, a total of 1908 households participated and 705 did not (household participation rate = 73.0%). One-thousand-three-hundred-and-thirteen (69.1%) participating households were in rural locations (2015 national census assignment). Supplementary Table S1 shows details of participating households and non-participating households. Participating households were more likely to be in urban locations (p < 0.001) and were closer to the nearest CHC when compared to non-participating households (p < 0.001).

**Fig. 2:**
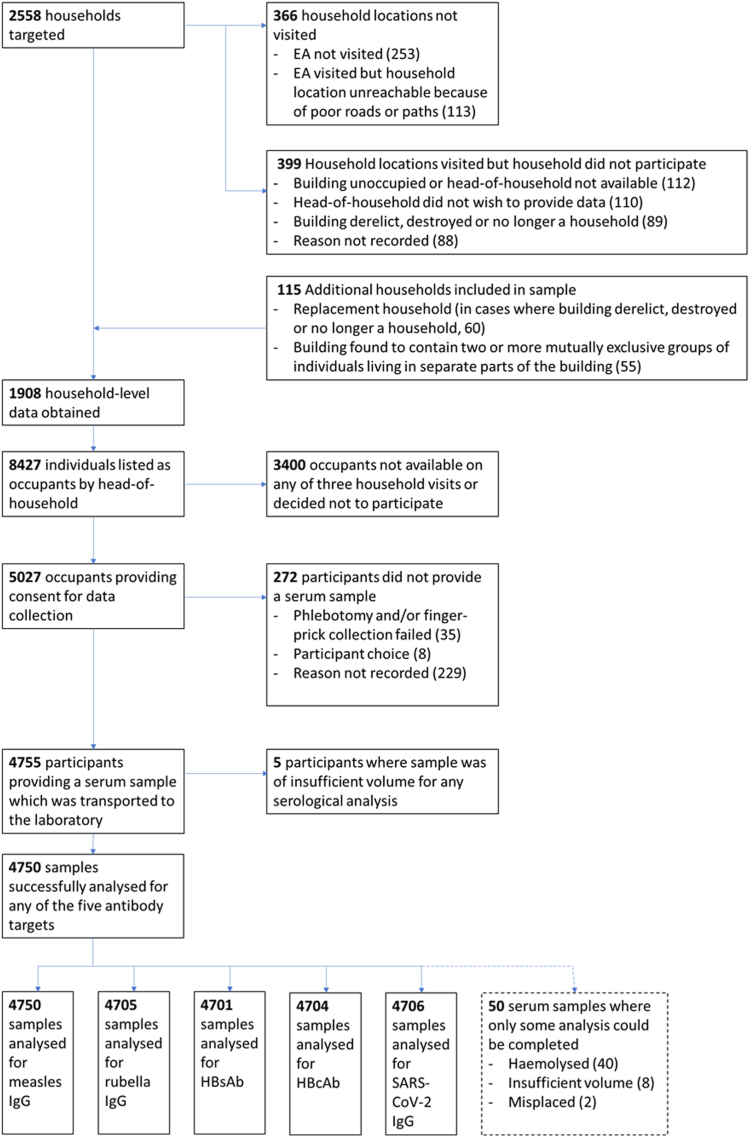
Flow diagram showing household- and individual-level participation in the study, and reasons for non-participation.

The total number of occupants within participating households was 8427. Of these, 4750 (56.4%) participated. Fig. 2 shows details of individual and household participation. Table 1 shows demographic details of participants and non-participating household occupants. Individual participation rate was significantly higher among females (62.6%) compared to males (50.6%, p < 0.001) and lower among children aged under 5 years (44.3%) compared to other age strata (p < 0.001).

**Table 1:** Summary of participants (N = 4750) and comparison to non-participating household occupants (N = 3677).

Individual-level characteristic	Participants (n = 4750)	Non-participating household occupants (N = 3677)	Total
Median age in years (IQR)	28 (13–46)	23 (10–41)	26 (12–44)
Age group (%)			
1–4 years	271 (5.7)	341 (9.3)	612 (7.3)
5–14 years	1039 (21.9)	637 (17.3)	1676 (19.9)
15–24 years	812 (17.1)	551 (15.0)	1363 (16.2)
25–40 years	1170 (24.6)	680 (18.5)	1850 (22.0)
41+ years	1458 (30.7)	737 (20.0)	2195 (26.0)
Not recorded	0 (0.0)	731 (19.9)	731 (8.7)
Adult or child (%)			
Child	1400 (29.5)	1299 (35.3)	2699 (32.0)
Adult	3350 (70.5)	2282 (62.1)	5632 (66.8)
Not recorded	0 (0.0)	96 (2.6)	96 (1.1)
Gender			
Male	1941 (40.9)	1896 (51.6)	3837 (45.5)
Female	2808 (59.1)	1677 (45.6)	4485 (53.2)
Other/unknown/not recorded	1 (0.0)	104 (2.8)	105 (1.2)
Median number of household occupants (IQR)	5 (4–7)	5 (4–7)	5 (4–7)
Total	4750	3677	8427

Abbreviation: IQR, interquartile range.

### Measles IgG seroprevalence

**Fig. 3:**
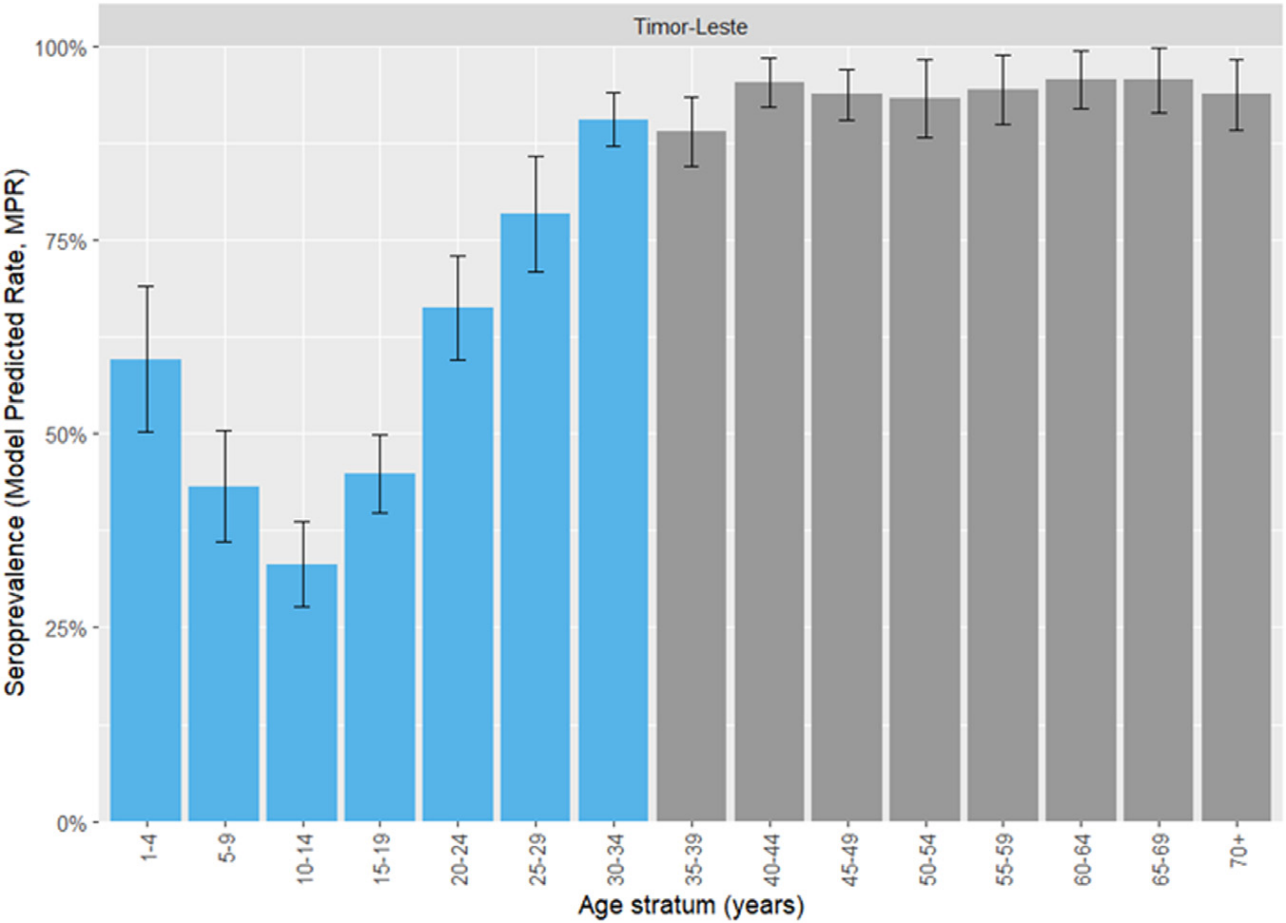
Model-predicted measles IgG seroprevalence estimates for the Timor-Leste general population. Vaccine-eligible age strata are shown in blue.

MPRs for the Timor-Leste general population (age-strata at 5-year intervals) are shown in Fig. 3 (municipality-level findings are shown in Supplementary Figure S1. Seroprevalence was lowest among adolescents aged 10–14 years (33.2%, 95% CI 27.8–38.6%). It increased in younger children to 59.6% (95% CI 50.2–69.0%) among those aged 1–4 years. In adults, seroprevalence increased with age and plateaued at >90% (central estimates) for all age strata >39 years.

National and municipal CPRs and MPRs in *a priori*-determined age-strata and the measles vaccine-eligible stratum (1–34 years) are shown in Table 2. Seroprevalence in the vaccine-eligible age stratum varied between municipalities, being highest in Dili (72.1%, 95% CI 66.7–77.5%) and lowest in Lautem (37.0%, 95% CI 22.9–51.0%).

**Table 2:** National and municipal measles IgG seroprevalence estimates (model-predicted rates, MPRs).

	1–4 years			5–14 years			15–24 years			25–39 years			40+ years			All age groups			Vaccine-eligible (1–34 years)		
	n	CPR (%)	MPR (%)	n	CPR (%)	MPR (%)	n	CPR (%)	MPR (%)	n	CPR (%)	MPR (%)	n	CPR (%)	MPR (%)	n	CPR (%)	MPR (%)	n	CPR (%)	MPR (%)
Aileu	0	–	–	33	33.3	33.1 (23.1–43.1)	40	45.0	48.9 (39.4–58.3)	45	93.3	83.2 (77.3–89.2)	63	85.7	93.3 (89.7–96.8)	181	69.1	69.3 (62.3–76.3)	108	56.5	54.2 (44.9–63.6)
Ainaro	18	44.4	51.4 (41.6–61.3)	79	25.3	31.2 (25.8–36.6)	49	44.9	47.1 (39.5–54.8)	53	83.0	82.2 (77.3–87.1)	86	96.5	92.8 (90.4–95.2)	285	62.1	64.2 (61.3–67.0)	191	46.1	50.3 (46.6–53.9)
Baucau	28	67.9	65.8 (56.5–75.1)	78	29.4	46.0 (38.6–53.5)	61	72.1	61.9 (55.2–68.7)	106	89.6	90.3 (87.1–93.4)	168	98.2	96.6 (95.3–97.9)	441	78.5	79.3 (76.5–82.2)	242	62.4	66.2 (62.0–70.5)
Bobonaro	14	21.4	42.6 (31.4–53.8)	79	13.9	25.1 (18.8–31.5)	50	36.0	39.3 (32.3–46.3)	101	82.2	77.2 (70.7–83.7)	135	90.4	90.0 (86.4–93.7)	379	62.5	66.4 (61.2–71.6)	218	41.7	47.9 (41.4–54.4)
Covalima	27	37.0	53.2 (42.2–64.2)	80	36.3	34.2 (27.5–50.0)	41	51.2	50.0 (42.5–57.6)	86	84.9	84.3 (79.7–88.8)	120	93.3	93.8 (91.5–96.1)	354	69.2	68.0 (63.8–72.1)	205	52.7	52.5 (47.0–58.1)
Dili	64	54.7	72.9 (62.6–83.3)	264	46.7	54.5 (46.0–63.1)	299	68.6	69.7 (63.0–76.3)	305	90.2	93.5 (90.8–96.2)	258	98.1	97.8 (96.7–98.9)	1200	74.7	79.8 (75.8–83.8)	844	65.1	72.1 (66.7–77.5)
Ermera	19	42.1	57.3 (41.8–72.7)	60	35.0	38.6 (26.5–50.6)	58	50.0	53.4 (41.8–65.1)	86	86.0	86.4 (79.9–92.9)	104	93.3	94.7 (91.6–97.8)	327	70.0	72.9 (65.6–80.3)	199	55.3	59.2 (49.3–69.2)
Lautem	16	56.3	45.1 (25.1–65.0)	94	42.6	25.3 (11.2–39.5)	26	46.2	40.6 (25.0–56.1)	40	77.5	77.6 (65.9–89.3)	140	84.3	90.2 (82.4–98.1)	316	66.5	60.6 (49.3–71.8)	159	49.7	37.0 (22.9–51.0)
Liquiçá	15	66.7	60.1 (45.3–74.9)	33	30.3	38.3 (27.0–49.7)	37	56.8	54.6 (44.5–64.7)	62	87.1	87.1 (80.9–93.3)	78	96.2	95.2 (92.5–98.0)	225	75.6	76.2 (69.9–82.4)	123	70.0	61.0 (52.2–69.7)
Manatuto	24	69.6	59.9 (47.0–72.8)	63	39.7	38.2 (28.2–48.2)	28	53.6	54.8 (44.4–65.2)	53	83.0	86.9 (81.1–92.8)	80	97.5	95.4 (92.9–97.9)	247	72.1	72.6 (66.6–78.7)	149	56.4	57.7 (49.1–66.2)
Manufahi	20	65.0	67.4 (58.0–76.8)	38	31.6	47.5 (39.5–55.5)	24	58.3	59.2 (52.4–66.0)	40	82.5	89.7 (85.7–93.7)	54	100	96.9 (95.5–98.4)	176	71.6	76.9 (73.1–80.7)	111	55.0	64.9 (59.5–70.3)
Oecusse	15	53.3	68.0 (55.0–81.1)	62	41.9	47.8 (37.4–60.1)	42	69.0	64.7 (54.1–75.3)	55	90.1	90.7 (85.6–95.7)	124	98.4	96.9 (95.0–98.9)	298	78.9	79.5 (73.7–85.2)	155	61.9	63.8 (54.7–73.0)
Viqueque	12	50.0	52.4 (39.9–64.8)	66	39.4	33.0 (25.7–40.4)	57	56.1	49.8 (41.5–58.1)	75	82.7	81.8 (75.4–88.2)	111	91.9	93.2 (89.9–96.5)	321	71.0	70.0 (64.9–75.2)	187	56.2	53.7 (47.0–60.4)
Timor-Leste	271	53.5	59.6 (50.2–69.0)	1039	36.8	38.3 (33.3–43.3)	812	59.1	55.9 (52.2–59.6)	1107	86.7	86.2 (83.4–89.0)	1521	94.3	94.5 (92.7–96.2)	4750	71.6	72.8 (71.0–74.5)	2891	57.4	58.6 (56.2–60.9)

Abbreviations: n, number of participants; CPR, crude positive rate; MPR, model-predicted rate.

In addition to ‘age stratum’ and ‘municipality of residence’, seropositivity in vaccine-eligible individuals was negatively associated with ‘male gender’ (adjusted OR = 0.645, p = 0.001, see Table 3 and Supplementary Table S2).

**Table 3:** Results from multivariable mixed-effects logistic regression analyses assessing whether individual and household characteristics were associated with seropositivity for vaccine-preventable diseases (VPDs).

	Measles IgG (N = 2891)			Rubella IgG (N = 801)			ScV2 anti-S IgG (N = 3416)			HBsAb (N = 1290)			HBcAb (N = 1290)		
	OR	(95% CI)	p value	OR	(95% CI)	p value	OR	(95% CI)	p value	OR	(95% CI)	p value	OR	(95% CI)	p value
**Fixed effects**															
Age stratum															
1–4 years	ref	ref	ref	ref	ref	ref	–	–	–	ref	ref	ref	ref	ref	ref
5–9 years	0.34	(0.16–0.73)	0.006	2.10	(0.79–5.55)	0.135	–	–	–	0.34	(0.16–0.74)	0.007	13.14	(0.26–6685.54)	0.414
10–14 years	0.17	(0.08–0.38)	<0.001	–	–	–	–	–	–	0.05	(0.02–0.13)	<0.001	97.25	(0.04–217359.00)	0.241
15–19 years	0.33	(0.16–0.72)	0.005	–	–	–	ref	ref	ref	–	–	–	–	–	–
20–24 years	1.35	(0.49–3.73)	0.564	–	–	–	0.14	(0.03–0.68)	0.016	–	–	–	–	–	–
25–29 years	3.69	(1.28–10.70)	0.017	–	–	–	0.11	(0.02–0.70)	0.020	–	–	–	–	–	–
30–34 years	16.44	(6.91–39.11)	<0.001	–	–	–	0.23	(0.03–1.67)	0.146	–	–	–	–	–	–
35–39 years	–	–	–	–	–	–	0.15	(0.02–1.05)	0.056	–	–	–	–	–	–
40–44 years	–	–	–	–	–	–	0.24	(0.04–1.30)	0.096	–	–	–	–	–	–
45–49 years	–	–	–	–	–	–	0.13	(0.02–0.76)	0.024	–	–	–	–	–	–
50–54 years	–	–	–	–	–	–	1.19	(0.11–13.51)	0.887	–	–	–	–	–	–
55–59 years	–	–	–	–	–	–	0.07	(0.01–0.52)	0.011	–	–	–	–	–	–
60–64 years	–	–	–	–	–	–	0.18	(0.02–1.67)	0.129	–	–	–	–	–	–
65–69 years	–	–	–	–	–	–	0.01	(0.00–0.06)	<0.001	–	–	–	–	–	–
70+ years	–	–	–	–	–	–	0.04	(0.01–0.27)	0.001	–	–	–	–	–	–
Gender															
Female	ref	ref	ref	ref	ref	ref	ref	ref	ref	ref	ref	ref	ref	ref	ref
Male	0.65	(0.48–0.87)	0.005	1.20	(0.43–2.84)	0.843	0.60	(0.31–1.18)	0.136	1.37	(0.84–2.25)	0.203	5.67	(0.73–44.23)	0.996
HH occupancy	1.01	(0.95–1.08)	0.701	0.87	(0.67–0.13)	0.287	0.95	(0.77–1.17)	0.628	1.06	(0.91–1.22)	0.462	1.09	(0.56–2.151)	0.797
Recent fever	–	–	–	–	–	–	1.19	(0.27–5.18)	0.814	–	–	–	–	–	–
SARS-CoV-2 vaccination	–	–	–	–	–	–	55.85	(14.5–214.54)	<0.001	–	–	–	–	–	–
Day of outbreak	–	–	–	–	–	–	1.01	(1.00–1.03)	0.079	–	–	–	–	–	–
Municipality															
Dili	ref	ref	ref	ref	ref	ref	ref	ref	ref	ref	ref	ref	ref	ref	ref
Aileu	0.25	(0.08–0.82)	0.023	5.79	(0.01–7104.57)	0.625	0.30	(0.03–3.11)	0.307	2.27	(0.15–34.23)	0.550	0.86	(0.00–2.73 × 10^23^)	0.996
Ainaro	0.17	(0.06–0.43)	<0.001	0.01	(0.00–0.12)	0.001	3.17	(0.05–202.86)	0.582	0.74	(0.15–3.74)	0.717	0.56	(0.00–2.28 × 10^8^)	0.953
Baucau	0.13	(0.15–0.96)	0.040	7.23	(0.11–474.37)	0.350	18.95	(0.11–3377.28)	0.262	0.44	(0.06–3.46)	0.427	1.94	(0.00–4.97 × 10^6^)	0.929
Bobonaro	0.05	(0.02–0.18)	<0.001	0.03	(0.00–2.79)	0.127	2.84	(0.13–63.26)	0.507	2.45	(0.19–32.07)	0.490	0.05	(0.00–1145.47)	0.553
Covalima	0.19	(0.06–0.59)	0.004	0.00	(0.00–0.62)	0.023	1.61	(0.06–43.78)	0.776	2.00	(0.24–16.64)	0.516	0.94	(0.00–2.82 × 10^14^)	0.997
Ermera	0.30	(0.10–0.84)	0.023	0.50	(0.01–23.30)	0.720	1.49	(0.05–49.11)	0.820	3.64	(0.54–24.51)	0.182	3.32	(0.00–3.66 × 10^5^)	0.838
Lautem	0.18	(0.05–0.68)	0.012	0.13	(0.00–60.67)	0.507	2.08	(0.07–58.67)	0.663	0.46	(0.05–3.89)	0.470	25.32	(0.00–1.14 × 10^13^)	0.811
Liquiçá	0.39	(0.14–1.10)	0.075	1.50	(0.12–179.78)	0.868	8.83	(0.35–220.26)	0.182	1.15	(0.05–26.35)	0.929	0.13	(0.00–3564.29)	0.694
Manatuto	0.24	(0.06–0.91)	0.036	0.26	(0.01–10.28)	0.469	3.12	(0.05–218.12)	0.596	1.95	(0.32–11.93)	0.466	13.80	(0.00–7.77 × 10^8^)	0.771
Manufahi	0.31	(0.12–0.82)	0.019	0.12	(0.00–23.65)	0.431	15.59	(0.05–4894.98)	0.345	1.25	(0.22–7.27)	0.802	6.13	(0.00–1.47 × 10^7^)	0.807
Oecusse	0.53	(0.19–1.52)	0.235	0.09	(0.01–1.20)	0.068	0.87	(0.01–156.82)	0.956	0.50	(0.11–2.32)	0.376	3.56	(0.00–8.69 × 10^7^)	0.882
Viqueque	0.21	(0.08–0.56)	0.002	0.61	(0.01–26.90)	0.795	2.25	(0.07–70.85)	0.642	0.85	(0.09–8.03)	0.884	1.66	(0.00–1.95 × 10^16^)	0.978
Log (dist to CHC)	1.33	(0.94–1.88)	0.109	1.30	(0.35–4.85)	0.695	1.93	(0.98–3.79)	0.057	1.17	(0.85–1.63)	0.335	1.89	(0.13–27.55)	0.797
Log (EA pop density)	0.98	(0.83–1.15)	0.787	1.74	(0.75–4.03)	0.196	0.70	(0.37–1.30)	0.255	1.36	(0.86–2.17)	0.187	0.47	(0.02–12.51)	0.645
Household location															
Rural	ref	ref	ref	ref	ref	ref	ref	ref	ref	ref	ref	ref	ref	ref	ref
Urban	1.25	(0.55–2.86)	0.588	0.04	(0.00–0.39)	0.007	99.67	(4.25–2338.61)	0.005	0.23	(0.49–1.08)	0.062	9.46	(0.02–4960.03)	0.478
**Random effects**															
Enumeration area	0.79	(0.44–1.44)	–	13.04	(5.85–29.08)	–	10.15	(4.93–20.92)	–	3.55	(1.69–7.46)	–	20.97	(2.28–193.09)	–
Household	3.35	(2.33–4.82)	–	9.149	(4.07–20.57)	–	19.13	(8.00–45.76)	–	6.19	(3.68–10.40)	–	17.06	(0.20–1428.31)	–

### Rubella IgG seroprevalence

**Fig. 4:**
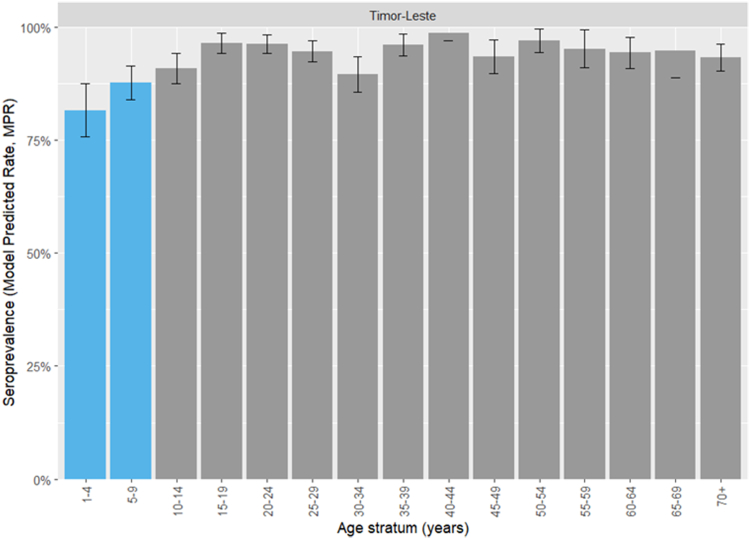
Model-predicted rubella IgG seroprevalence estimates for the Timor-Leste general population. Vaccine-eligible age strata are shown in blue.

MPRs for the Timor-Leste general population (age-strata at 5-year intervals) are shown in Fig. 4 (municipality-level findings are shown in Supplementary Figure S2). Seroprevalence was lowest among children aged 1–4 (81.6%, 95% CI 75.8–87.5%). It increased with age and plateaued >89% (central estimates) for all age strata >9 years.

National and municipal CPRs and MPRs in *a priori*-determined age-strata and the rubella vaccine-eligible age stratum are shown in Table 4.

**Table 4:** National and municipal rubella IgG seroprevalence estimates (model-predicted rates, MPRs).

	1–4 years			5–14 years			15–24 years			25–39 years			40+ years			All age groups			Vaccine-eligible (1–9 years)		
	n	CPR (%)	MPR (%)	n	CPR (%)	MPR (%)	n	CPR (%)	MPR (%)	n	CPR (%)	MPR (%)	n	CPR (%)	MPR (%)	n	CPR (%)	MPR (%)	n	CPR (%)	MPR (%)
Aileu	0	–	–	33	81.8	87.3 (77.2–97.4)	40	95	95.4 (90–101)	45	93.3	92.1 (85.1–99.2)	63	96.8	94.9 (90.1–99.7)	181	92.8	93.1 (86.9–99.3)	19	94.7	85.2 (73.9–96.5)
Ainaro	18	50	72.7 (62.1–83.3)	79	78.5	83.6 (77.1–90.1)	49	98	93.7 (90.6–96.8)	53	98.1	89.2 (84.7–93.7)	86	91.9	92.2 (88.8–95.7)	285	87.7	88.6 (84.5–92.7)	59	64.4	78.8 (70.8–86.7)
Baucau	3	100	89.3 (80.3–98.2)	12	91.7	94.5 (89.4–99.7)	11	90.9	98.4 (96.4–100)	17	88.2	96.3 (92.6–100)	26	84.6	97.7 (95.3–100)	441	94.3	96.5 (93.2–99.8)	67	94	91.7 (84.6–98.8)
Bobonaro	13	46.2	63.4 (49.8–77)	79	82.3	76.3 (66.2–86.5)	50	94	89.5 (83.5–95.5)	101	81.2	82.4 (75.5–89.4)	135	92.6	87.6 (82.3–93)	378	86.0	84.1 (77.8–90.3)	53	67.9	70.3 (59.1–81.5)
Covalima	27	70.4	76.1 (66.3–85.9)	80	91.3	85.6 (81–90.3)	41	92.7	94.7 (91.6–97.8)	86	89.5	91.1 (87.6–94.6)	119	94.1	93.0 (90–95.9)	353	90.4	89.5 (86.2–92.9)	71	80.3	80.9 (74.5–87.3)
Dili	60	88.3	84.4 (77.8–91.1)	272	90.4	91.6 (88.2–94.9)	298	95.6	97.2 (95.6–98.9)	305	94.8	94.8 (92.5–97.1)	258	95.7	96.6 (94.9–98.4)	1193	93.9	94.9 (93.0–96.8)	200	88.5	88.7 (84.2–93.2)
Ermera	19	78.9	83.6 (72.9–94.4)	60	98.3	91 .0 (85.1–96.9)	58	94.8	97.1 (94.3–99.8)	86	93	93.8 (89.3–98.3)	104	97.1	96.4 (93.7–99)	327	93.3	94.2 (90.3–98.0)	46	89.1	86.8 (78.4–95.2)
Lautem	14	85.7	83.2 (72.3–94)	93	91.4	90.9 (84.5–97.4)	26	100	97 (94–99.9)	40	95	95.0 (90.8–99.2)	139	93.5	96.4 (93.1–99.6)	312	94.2	94.2 (89.8–98.5)	64	90.6	88.3 (79.7–96.9)
Liquiçá	15	86.7	83.7 (77.1–90.2)	33	87.9	91.3 (87.7–94.9)	37	100	97.2 (95.6–98.8)	62	96.8	95.1 (92.9–97.3)	78	93.6	96.0 (93.8–98.1)	225	91.5	94.9 (92.9–96.8)	31	87.1	87.5 (82.7–92.2)
Manatuto	21	81	75.3 (68.8–81.9)	60	90	85.8 (82.4–89.2)	27	100	94.5 (92.1–96.9)	51	88.2	90.8 (87.7–94)	77	94.8	93.2 (91–95.5)	236	92.6	89.7 (87.8–91.6)	48	83.3	79.5 (75–84)
Manufahi	20	75	81.9 (68.9–95)	38	92.1	89.8 (81.8–97.7)	24	100	96.7 (93.2–100)	40	92.5	94.0 (88.5–99.4)	54	96.3	95.5 (91.2–99.8)	176	94.6	92.9 (87.2–98.6)	41	80.5	85.7 (75.3–96)
Oecusse	15	73.3	83.7 (77.8–89.5)	62	91.9	90.8 (88–93.6)	42	100	97.1 (95.6–98.6)	55	96.4	94.8 (92.8–96.7)	124	96	96.4 (94.9–97.8)	298	95.3	94.3 (92.7–95.8)	53	83	88.2 (84.7–91.7)
Viqueque	10	80	86.7 (77.9–95.5)	64	93.8	92.8 (88–97.6)	51	100	97.8 (95.9–99.6)	71	95.8	95.9 (92.7–99.1)	104	95.2	96.8 (94.5–99.2)	300	93.9	95.8 (92.9–98.6)	49	87.8	90.4 (84.2–96.6)
Timor-Leste	260	78.5	81.6 (75.8–87.5)	1031	89.3	89.3 (86.9–91.7)	804	96.6	96.4 (94.8–98.0)	1101	93	93.3 (91.5–95.0)	1509	94.9	95.3 (94–96.5)	4705	92.6	93.2 (92.2–94.2)	801	84.3	85.9 (82.4–89.3)

Abbreviations: n, number of participants; CPR, crude positive rate; MPR, model-predicted rate.

### SARS-CoV-2 anti-S IgG seroprevalence

**Fig. 5:**
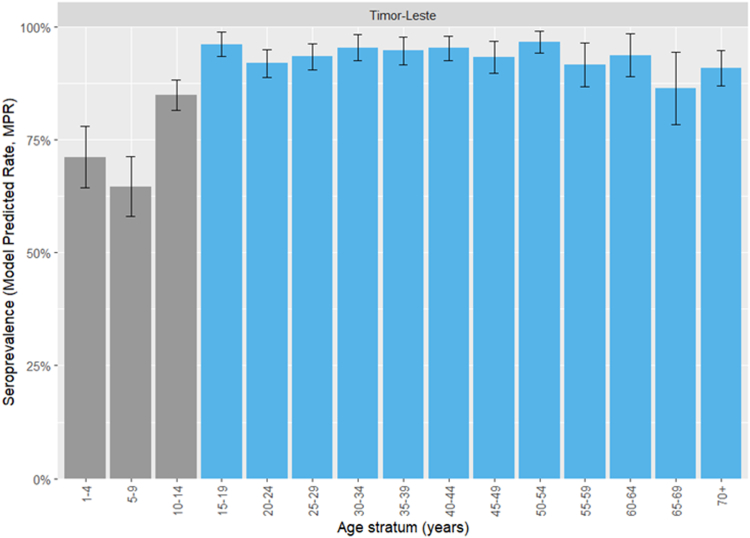
Model-predicted severe acute respiratory syndrome coronavirus 2 (SARSCoV-2) anti-spike IgG seroprevalence estimates for the Timor-Leste general population.

MPRs for the Timor-Leste general population (age-strata at 5-year intervals) are shown in Fig. 5 (municipality-level findings are shown in Supplementary Figure S3). Seroprevalence was lowest among children aged 1–4 years (71.2%, 95% CI 64.4–78.0%) and 5–9 years (64.7%, 95% CI 58.1–71.3%). It increased with age and plateaued >91% (central estimates) for all age strata between 15 and 64 years. Seroprevalence was lower among adults aged 65–69 years (86.4, 95% CI 78.5–94.4%) and 70+ (90.1%, 95% CI 86.9–94.8%) compared to other adults.

National and municipal CPRs and MPRs in *a priori*-determined age-strata and the SARS-CoV-2 vaccine-eligible group are shown in Table 5. Seroprevalence in the vaccine-eligible age stratum varied between municipalities, being highest in Baucau (98.0%, 95% CI 96.6–99.4%) and lowest in Aileu (86.7%, 95% CI 80.8–92.6%).

**Table 5:** National and municipal severe acute respiratory syndrome coronavirus 2 (SARS-CoV-2) anti spike protein (anti-S) IgG seroprevalence estimates (model-predicted rates, MPRs).

	1–4 years			5–14 years			15–24 years			25–39 years			40+ years			All age groups			Vaccine-eligible (>14 years)		
	n	CPR (%)	MPR (%)	n	CPR (%)	MPR (%)	n	CPR (%)	MPR (%)	n	CPR (%)	MPR (%)	n	CPR (%)	MPR (%)	n	CPR (%)	MPR (%)	n	CPR (%)	MPR (%)
Aileu				33	57.6	59.5 (48.8–70.2)	40	92.5	88.3 (82.6–94.1)	45	95.6	87.3 (80.9–93.6)	63	79.4	85 (77.9–92.1)	181	82.3	82.4 (75.9–88.9)	148	87.8	86.7 (80.8–92.6)
Ainaro	18	77.8	72.6 (64.5–80.7)	79	74.7	76 (70.9–81.2)	49	93.9	95.4 (93.1–97.6)	53	96.2	95.4 (93–97.8)	86	95.3	93.9 (91.5–96.4)	285	88.4	88.8 (86.0–91.5)	188	95.2	94.8 (92.7–96.8)
Baucau	28	82.1	84.5 (76.3–92.7)	78	85.9	87.5 (81.7–93.4)	61	100	98.3 (96.9–99.6)	106	98.1	98.3 (97–99.7)	168	97.6	97.7 (96–99.3)	441	95	95.5 (93.2–97.9)	335	98.2	98 (96.6–99.4)
Bobonaro	14	64.3	57.9 (45.7–70.1)	79	65.8	68.5 (60.6–76.4)	50	90	89.7 (84.5–94.9)	101	92.1	91.1 (86.6–95.7)	135	89.6	88.8 (84.2–93.4)	379	84.4	85.3 (80.4–90.1)	286	90.6	89.7 (85.5–94)
Covalima	27	70.4	68.3 (62.2–74.4)	80	65	74.2 (68.8–79.6)	41	90.2	93.6 (90.7–96.5)	86	97.7	94.8 (92.6–97)	120	100	92.8 (90.4–95.1)	354	88.1	86.8 (84.3–89.3)	247	97.6	93.7 (91.8–95.6)
Dili	60	68.3	67.9 (58.7–77)	272	78.3	74.2 (69–79.5)	298	96	93.9 (91.2–96.5)	305	91.8	94.5 (91.9–97.2)	258	89.5	93.6 (91.1–96.2)	1193	88.1	89.4 (86.8–92.1)	861	92.6	94 (91.9–96.2)
Ermera	19	68.4	72.4 (61.1–83.8)	60	86.7	80.3 (72.7–87.9)	58	98.3	95.6 (92.7–98.5)	86	96.5	96 (92.9–99.1)	104	91.3	94.5 (90.9–98.1)	327	91.7	91.4 (87.4–95.5)	248	94.8	95.3 (92.2–98.3)
Lautem	14	42.9	68.6 (50.3–87)	92	77.2	73.4 (59.7–87.1)	26	92.3	94.1 (89–99.2)	40	92.5	94.4 (89.4–99.3)	140	95	93.4 (88.1–98.8)	312	86.9	86.7 (78.8–94.7)	206	94.2	93.8 (88.7–98.8)
Liquiçá	15	66.7	75.3 (51.8–98.9)	33	72.7	78.7 (57.8–99.7)	37	97.3	95.7 (89–103)	62	88.7	96.2 (89.7–103)	78	91	94.1 (85.2–103)	225	87.1	92.5 (82.8–100.0)	177	91.5	95.3 (87.9–103)
Manatuto	21	66.7	65.1 (52.1–78.2)	59	71.2	72.8 (63.1–82.5)	27	96.3	93.6 (89.2–98)	51	98	93.8 (89.7–98)	77	93.5	92.3 (88.1–96.6)	235	86.8	86.4 (80.8–92.1)	155	95.5	93.1 (89.1–97.1)
Manufahi	20	85	73.7 (61.1–86.3)	38	65.8	77.4 (66.5–88.2)	24	100	96 (92.6–99.4)	40	97.5	96.2 (93–99.4)	54	96.3	95 (91.2–98.8)	176	89.2	89.9 (84.5–95.4)	118	97.5	95.6 (92.3–99)
Oecusse	15	46.7	70.4 (55.5–85.2)	62	72.6	73.9 (62.8–85)	42	95.2	94.4 (90.5–98.3)	55	96.4	95.3 (91.6–98.9)	124	96.8	93.7 (89.2–98.1)	298	88.9	88.4 (82.5–94.3)	221	96.4	94.2 (90.3–98.2)
Viqueque	10	40	62.2 (49.7–74.7)	64	60.9	67.7 (59.5–76)	51	96.1	92.2 (89.1–95.3)	71	94.4	92.5 (88.3–96.7)	104	98.1	90.1 (86–94.2)	300	87	86.2 (81.9–90.5)	226	96.5	91.3 (87.9–94.8)
Timor-Leste	261	67.8	71.2 (64.4–78)	1029	73.9	74.6 (71–78.2)	804	95.5	94 (92.1–95.9)	1101	94.4	94.6 (92.4–96.8)	1511	93.5	93 (91.1–94.9)	4706	88.3	88.9 (87.4–90.4)	861	94.3	93.8 (92.3–95.2)

Abbreviations: n, number of participants; CPR, crude positive rate; MPR, model-predicted rate.

In addition to ‘age stratum’ and ‘municipality of residence’, seropositivity in vaccine-eligible individuals was also associated with ‘urban household location’ (adjusted OR = 99.67, p = 0.005) and ‘previous receipt of vaccination against COVID-19’ (adjusted OR = 55.85, p < 0.001, see Table 3 and Supplementary Table S4).

### HBsAb seroprevalence

**Fig. 6:**
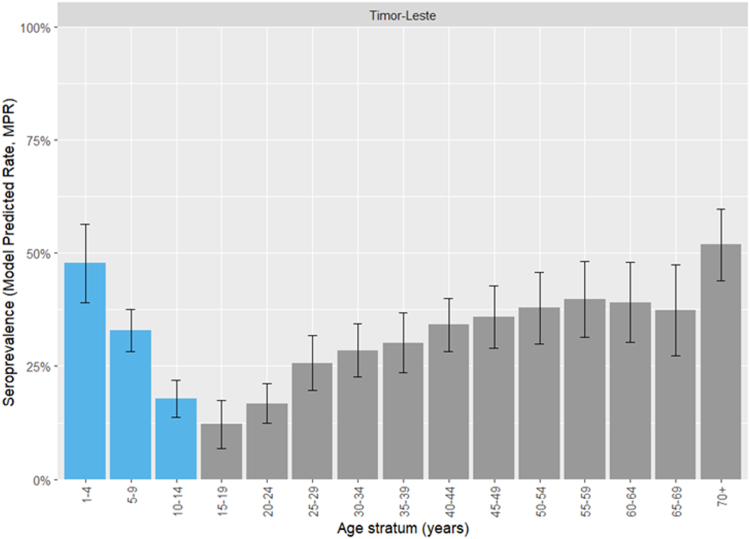
Model-predicted hepatitis B surface antibody (HBsAb) seroprevalence estimates for the Timor-Leste general population. Vaccine-eligible age strata are.

MPRs for the Timor-Leste general population (age-strata at 5-year intervals) are shown in Fig. 6 (municipality-level findings are shown in Supplementary Figure S4). Seroprevalence was lowest among adolescents aged 15–19 years (12.1%, 95% CI 6.8–17.5%). It increased in younger age groups to 47.8% (95% CI 39.0–56.5%) among children aged 1–4 years. It also increased in older age strata, to 51.9% (95% CI 44.0–59.8%) among those aged 70+ years.

National and municipal CPRs and MPRs in *a priori*-determined age-strata and the hepatitis B vaccine-eligible group are shown in Table 6. Seroprevalence in the vaccine-eligible age stratum varied between municipalities, being highest in Manufahi (39.6%, 95% CI 25.6–53.6%) and lowest in Aileu (17.5%, 95% CI 12.4–22.6%).

**Table 6:** National and municipal hepatitis B surface antibody (HBsAb) seroprevalence estimates (model-predicted rates, MPRs).

	1–4 years			5–14 years			15–24 years			25–39 years			40+ years			All age groups			Vaccine-eligible (1–14 years)		
	n	CPR (%)	MPR (%)	n	CPR (%)	MPR (%)	n	CPR (%)	MPR (%)	n	CPR (%)	MPR (%)	n	CPR (%)	MPR (%)	n	CPR (%)	MPR (%)	n	CPR (%)	MPR (%)
Aileu				33	30.3	17.5 (12.4–22.6)	40	12.5	8.85 (5.56–12.1)	45	15.6	20.3 (14.9–25.6)	63	28.6	30.2 (24.3–36.1)	181	22.1	20.3 (16.1–24.4)	33	30.3	17.5 (12.4–22.6)
Ainaro	18	38.9	48.5 (34.5–62.5)	79	26.6	27.6 (18.3–36.9)	49	10.2	15.1 (8.35–21.9)	53	43.4	29.5 (18.9–40)	86	34.9	43.1 (31.7–54.5)	285	30.2	31.5 (22.3–40.7)	97	28.9	31.5 (21.9–41.2)
Baucau	28	21.4	42.7 (31.7–53.7)	78	16.7	21.8 (16–27.5)	61	6.6	11.7 (7.01–16.4)	106	29.2	25 (18.5–31.6)	168	35.7	35 (28.3–41.8)	441	25.9	27.2 (21.7–32.6)	106	17.9	27.6 (21.2–34)
Bobonaro	13	38.5	50.1 (38.5–61.8)	79	16.5	24.1 (16.9–31.3)	50	20	15.6 (9.83–21.4)	101	22.8	29.2 (21.4–37)	135	37.8	41.4 (33.4–49.5)	378	27	31.5 (24.7–38.3)	92	19.6	26.5 (19.2–33.7)
Covalima	27	48.1	51.6 (40.5–62.7)	80	28.8	28.2 (20.5–35.8)	41	12.2	17.2 (11–23.5)	86	29.1	31.3 (22.7–39.9)	119	42.9	45.6 (36.8–54.4)	353	33.1	34.6 (27.2–41.9)	107	33.6	33.5 (25.9–41.2)
Dili	60	55	52.2 (43.1–61.2)	272	22.8	28.4 (23.6–33.2)	298	16.4	17.3 (13–21.6)	304	29.6	31.7 (27.1–36.4)	256	41.8	41.7 (36.4–47)	1190	28.7	30.2 (27.1–33.2)	332	28.6	31.7 (27.1–36.4)
Ermera	19	63.2	46.9 (33.1–60.8)	60	26.7	22.3 (15.9–28.8)	58	10.3	13.8 (8.26–19.4)	86	18.6	27.7 (20.3–35)	104	36.5	38.9 (30.4–47.5)	327	26.9	29.0 (22.3–35.7)	79	35.4	28.2 (20.5–35.9)
Lautem	14	35.7	50.2 (37.8–62.6)	93	17.2	28.3 (21–35.6)	26	19.2	16.3 (9.94–22.6)	40	25	32 (22.3–41.8)	139	46	43.9 (35.1–52.6)	312	32.1	34.0 (26.6–41.5)	107	19.6	30.4 (23–37.8)
Liquiçá	15	73.3	39.5 (30–49.1)	33	24.2	20.6 (15.7–25.6)	37	2.7	11.3 (8.06–14.5)	62	19.4	23.7 (18.2–29.2)	78	32.1	34.3 (28–40.5)	225	25.3	24.9 (20.7–29.2)	48	39.6	25.4 (20.3–30.6)
Manatuto	21	47.6	49.2 (34.8–63.5)	59	27.1	25.6 (15.2–36)	27	7.4	14.7 (6.61–22.7)	51	37.3	29.1 (17.4–40.8)	77	39	40.7 (28.8–52.6)	235	32.8	31.9 (21.4–42.5)	80	32.5	32.2 (21.3–43.1)
Manufahi	20	35	55.8 (39.5–72)	38	34.2	33.2 (19.3–47)	24	8.3	19.2 (7.9–30.6)	40	32.5	34.1 (18.6–49.6)	54	48.1	50.3 (35.2–65.4)	176	34.7	38.7 (24.9–52.6)	58	34.5	39.6 (25.6–53.6)
Oecusse	15	26.7	41.3 (30.2–52.4)	62	19.4	21.9 (16–27.9)	42	16.7	11.7 (7.35–16)	55	29.1	23 (16.1–29.9)	124	31.5	35.6 (27.8–43.4)	298	26.2	27.4 (21.3–33.6)	77	20.8	25.1 (18.9–31.4)
Viqueque	10	40	48.8 (38.8–58.7)	64	18.8	26.8 (21.4–32.2)	51	13.7	14.9 (10.2–19.6)	71	18.3	29.1 (22.7–35.6)	104	44.2	42.1 (35.3–48.9)	300	27.3	31.1 (26.1–36.1)	74	21.6	29.8 (24.4–35.2)
Timor-Leste	260	45	47.7 (39–56.5)	1030	22.8	25.5 (22.1–28.9)	804	13.4	14.5 (11.2–17.8)	1100	27.1	28.1 (24–32.3)	1507	38.8	39.7 (36.3–43.1)	4701	28.6	29.9 (28.2–31.6)	1290	27.3	29.6 (26.2–32.9)

Abbreviations: n, number of participants; CPR, crude positive rate; MPR, model-predicted rate.

### HBcAb seroprevalence

**Fig. 7:**
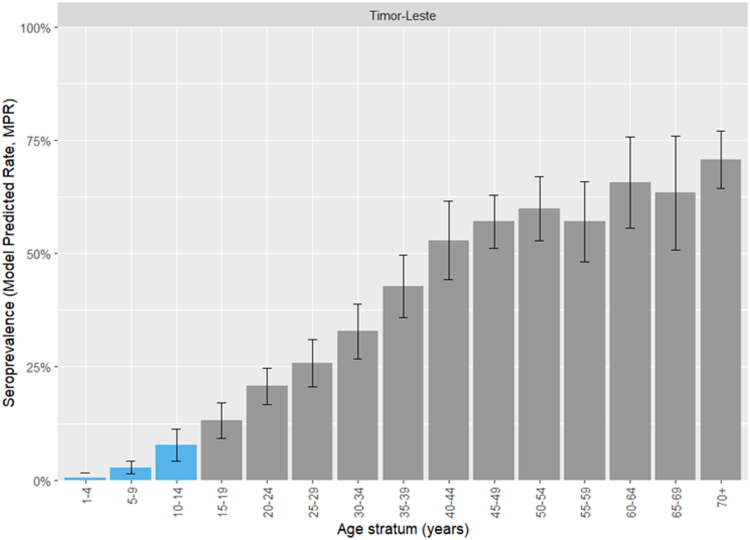
Model-predicted hepatitis B core antibody (HBcAb) seroprevalence estimates for the Timor-Leste general population. Vaccine-eligible age-strata are shown in blue.

MPRs for the Timor-Leste general population (age-strata at 5-year intervals) are shown in Fig. 7 (municipality-level findings are shown in Supplementary Figure S5). Seroprevalence was lowest among children aged 1–4 years (0.6%, 95% CI 0.0–1.5%). It increased as age strata increased, to 70.8% (95% CI 64.5–77.2%) among adults aged 70+ years.

National and municipal CPRs and MPRs in *a priori*-determined age-strata and the hepatitis B vaccine-eligible group are shown in Table 7.

**Table 7:** National and municipal hepatitis B core antibody (HBcAb) seroprevalence estimates (model-predicted rates, MPRs).

	1–4 years			5–14 years			15–24 years			25–39 years			40+ years			All age groups			Vaccine-eligible (1–14 years)		
	n	CPR (%)	MPR (%)	n	CPR (%)	MPR (%)	n	CPR (%)	MPR (%)	n	CPR (%)	MPR (%)	n	CPR (%)	MPR (%)	n	CPR (%)	MPR (%)	n	CPR (%)	MPR (%)
Aileu	0	–	–	33	3	2.65 (0.632–4.67)	40	12.5	10.1 (5.99–14.2)	45	24.4	24.9 (17.3–32.6)	63	49.2	50.0 (41.0–58.9)	181	26.5	26.0 (20.1–31.8)	33	3	2.65 (0.632–4.67)
Ainaro	18	0	0.279 (−0.276 to 0.833)	79	2.5	3.26 (0.987–5.53)	49	12.2	12.5 (6.07–18.9)	53	37.7	26.6 (16.9–36.3)	86	48.8	54.4 (42.5–66.4)	285	24.6	24.9 (17.9–31.9)	97	2.1	2.69 (0.827–4.56)
Baucau	28	0	0.511 (−0.424 to 1.45)	78	3.8	4.73 (2.07–7.4)	61	14.8	16.8 (10.9–22.7)	106	39.6	35.9 (27.3–44.5)	168	57.7	60.5 (51.9–69)	441	34.2	35.1 (29.0–41.2)	106	34.2	3.57 (1.6–5.53)
Bobonaro	13	0	0.444 (−0.411 to 1.3)	79	0	4.21 (1.32–7.09)	50	20	15.2 (8.83–21.6)	101	31.7	30 (20.7–39.3)	135	57	55.1 (44.9–65.3)	378	31.5	32.6 (25.1–40.0)	92	0	3.86 (1.22–6.5)
Covalima	27	0	0.469 (−0.425 to 1.36)	80	3.8	3.9 (1.34–6.47)	41	9.8	16.7 (9.85–23.5)	86	39.5	32.9 (23.4–42.5)	120	61.7	59.6 (50.0–69.3)	354	32.5	29.0 (22.7–35.3)	107	2.8	3.12 (1.09–5.15)
Dili	60	0	0.561 (−0.46 to 1.58)	272	2.6	4.84 (2.75–6.92)	298	18.5	18.4 (14.6–22.1)	305	37.4	34.7 (30.3–39.1)	257	56.8	58.8 (53.7–64)	1192	27	29.0 (26.3–31.7)	332	2.1	4.24 (2.44–6.03)
Ermera	19	0	0.247 (−0.217 to 0.711)	60	3.3	2.5 (1.24–3.76)	58	6.9	10.9 (7.89–14)	86	16.3	25.2 (20.9–29.6)	104	51.9	49.5 (45.2–53.7)	327	22.6	25.1 (22.8–27.5)	79	2.5	1.96 (1.01–2.92)
Lautem	14	0	1.24 (−0.909 to 3.38)	93	3.2	9.89 (6.49–13.3)	26	26.9	28.4 (21.7–35.1)	40	40	52.4 (44.1–60.8)	139	77.7	75.2 (69.9–80.5)	312	42.9	43.6 (39.3–47.8)	107	2.8	9.05 (5.96–12.1)
Liquiçá	15	0	0.204 (−0.187 to 0.595)	33	0	2.61 (1.33–3.89)	37	2.7	11.7 (8.93–14.5)	62	22.6	27.7 (22.9–32.5)	78	51.3	51.9 (44.1–59.8)	225	24.4	28.2 (24.2–32.2)	48	0	2.0 (1.04–2.96)
Manatuto	21	4.8	0.616 (−0.308 to 1.54)	59	6.8	6.04 (1.72–10.4)	27	14.8	17.7 (9.26–26.1)	51	41.2	36.4 (24.4–48.3)	77	58.4	61.9 (49.8–73.9)	235	31.9	32.9 (24.4–41.3)	80	6.3	4.52 (1.44–7.6)
Manufahi	20	0	0.827 (−0.693 to 2.35)	38	7.9	6.34 (2.2–10.5)	24	8.3	22.3 (13–31.5)	40	32.5	38.2 (27.0–49.4)	54	66.7	68.7 (59.3–78.1)	176	30.7	35.6 (28.1–43.2)	58	5.2	4.76 (1.67–7.85)
Oecusse	15	0	1.02 (−0.734 to 2.78)	62	3.2	6.7 (4.13–9.27)	42	28.6	26.1 (20–32.2)	55	47.3	44.6 (37.1–52.1)	124	71	71.7 (66.2–77.1)	298	43	42.7 (38.5–46.9)	77	2.6	5.76 (3.59–7.93)
Viqueque	10	0	0.777 (−0.576 to 2.13)	64	6.3	6.28 (3.64–8.93)	51	21.6	20.9 (16.1–25.8)	71	39.4	40.2 (33.8–46.6)	104	73.1	67.7 (62.0–73.4)	300	39.7	39.0 (34.8–43.1)	74	5.4	5.52 (3.24–7.8)
Timor-Leste	260	0.4	0.567 (−0.411 to 1.55)	1030	3.3	5.25 (3.39–7.1)	804	16.2	17.1 (14.2–19.9)	1101	35	33.7 (30.0–37.5)	1509	60.6	60.9 (57.8–64)	4704	31.1	32.5 (31.2–33.9)	1290	2.7	4.4 (2.9–5.9)

Abbreviations: n, number of participants; CPR, crude positive rate; MPR, model-predicted rate.

## Discussion

This national, population-representative serosurvey is among only a small number of studies conducted in low-middle income countries which have high participation and sampling suitable to estimate VPD seroprevalence across numerous age strata and regions, and assess association with key individual and household variables.^21^

A measles immunity gap was identified in Timor-Leste. This was most significant in individuals aged 10–14 years, in whom seroprevalence was only 33.2% (95% CI 27.8–38.6%). Low uptake of first and/or second doses of measles-containing vaccines in the absence of recent virus transmission, is likely the most important cause. Poor quality vaccines could also have contributed, for example if an effective cold chain could not been maintained.^22^^,^^23^ Compared to older children, those aged 1–4 years had higher measles IgG seroprevalence (59.6%, 95% CI 50.2–69.0%). This indicates an improvement in measles vaccine uptake in recent years. A vaccine coverage survey conducted in 2023 also found low rates of valid first (68.1%) and second (34.1%), doses of MR vaccine in Timorese children in their first and second year of life.^7^

High population immunity against measles is required to prevent transmission and WHO recommends that two-dose vaccine coverage should be 95% or above.^24^^,^^25^ In recent years there have been no confirmed cases of measles in Timor-Leste.^26^ However, data from this serosurvey suggest vaccination targets have not been achieved and a high potential for outbreaks among children and young adults. Since preliminary findings of this survey were reported to the Timor-Leste Ministry of Health, a national SIA was implemented during January to February 2023 which offered a single dose of MR vaccine to all children aged 9–59 months, achieving coverage of approximately 73.0%.^7^ SIAs targeting older children and adults should also be considered, in an attempt to increase overall population immunity and prevent measles outbreaks. Routine vaccination with MR at 9 and 18 months should also be strengthened.^27^

Rubella IgG seroprevalence was high among individuals aged over 9 years, who have not been eligible for rubella vaccination (first introduced in 2016). Therefore, virus transmission was likely occurring until at least 2016. Children aged 1–9 years (the vaccine-eligible group) had somewhat lower rubella seroprevalence. This ‘paradoxical’ observation may be due to a reduction in virus transmission having been achieved through partial population immunity, albeit with incomplete vaccine coverage. Rubella infections in children are often only mildly symptomatic or asymptomatic, and continued transmission is likely. It is also possible that higher seropositivity as compared with measles in vaccine eligible children could be due to a higher rate of seroconversion to the rubella component of the MR vaccine (than that to the measles component), particularly in individuals receiving only one dose.^28^ In June 2023, Timor-Leste received WHO verification of rubella elimination.^29^ Case-based surveillance of rubella (via testing of individuals with fever and rash) and surveillance for congenital rubella syndrome should be enhanced in Timor-Leste, particularly given that improved vaccine uptake in children could potentially result in upward age shifts in infection and transmission to females of childbearing age.

SARS-CoV-2 seroprevalence was high among individuals who were eligible for vaccination. Seroprevalence among children aged 1–14 years was also high (above 60%), despite this group being largely ineligible for vaccination at the time of this study. This finding is likely to indicate that widespread transmission of SARS-CoV-2 had occurred between March 2021 (onset of community transmission in Timor-Leste) and January 2023 (end of study data collection), as well as high vaccine uptake.^30^^,^^31^ Preliminary results from this study provided evidence, alongside data on disease epidemiology and severity, to support a decision not to introduce vaccination against SARS-CoV-2 for children aged <12 years in Timor-Leste.

A hepatitis B immunity gap was observed, indicated by low HBsAb seroprevalence in the vaccine-eligible population (children aged 1–14 years). This is likely due to incomplete coverage of hepatitis B vaccination, but could also relate to seroconversion failure and/or waning of HBsAb over time. However, similar to measles IgG seroprevalence, HBsAb seroprevalence is higher in younger children, indicating that there has been improvement in hepatitis B vaccine uptake in recent years. Seroprevalence of HBcAb, which is a marker associated with hepatitis B infection (but not vaccination), was very low among children aged 1–4 years, indicating that most children are being protected against vertical transmission of hepatitis B. It is evidence that Timor-Leste is moving towards its goal of eliminating mother-to-child transmission of hepatitis B by 2030.^32^

Among adults, HBcAb seroprevalence increased with age to >50% among those >40 years. This indicates high transmission of hepatitis B previously, and ongoing transmission among adolescents and young adults is likely. Findings are similar to those from other populations in the Southeast Asian region, including four provinces across Thailand (where seroprevalence in particpants >50 years was 60%) and four districts in Lao People's Democratic Republic (where seroprevalence in participants 31–40 years was 86.7%) Some of these individuals will have active (chronic) hepatitis B infection, putting them at risk of liver cirrhosis and hepatocellular carcinoma. A recent survey of healthcare workers in Timor-Leste also found a high rate of active hepatitis B infection.^8^ These findings highlight the need to strengthen services for evaluating and caring for individuals with active hepatitis B in Timor-Leste. Screening of all blood donors in Timor-Leste for hepatitis B is imperative. Testing should also be offered as part of screening for sexually transmitted infections, including during pregnancy.^33^

There were limitations to the study. It estimated seroprevalence of four priority VPDs in Timor-Leste but did not provide data on other relevant VPDs (diptheria, tetanus, pertussis, Haemophilus influenzae type B, polio and rotavirus). Household and individual participation rates were lower than anticipated at 73.0% and 56.4%, respectively, which may have reduced the precision and/or accuracy of seroprevalence estimates. Rural households were less likely to participate, and eight EAs (7.1%) could not be reached due to ‘poor weather and associated road damage’. Individuals in these communities may experience difficulty accessing healthcare including vaccination and therefore the study results may be biased towards higher estimates of VPD seroprevalence. The participation rate of young children (aged 1–4 years) was lower than the *a priori* target sample size.^12^ While observed demographic differences were accounted for by assigning sampling weights to each individual which were a factor of ‘selection probability’ and ‘response probability’ at each level, it is possible that other (unobserved) bias was not accounted for. Collecting additional data on non-participating households and individuals and further post-stratification adjustment or data raking could have further improved estimates.^34^^,^^35^ Similarly, when assessing and quantifying potential determinants of seropositivity, there was at times considerable uncertainty (wide confidence intervals) due to a small number of negative cases (e.g. SARS-CoV-2 IgG) or positive cases (e.g. HBcAb) among vaccine-eligible participants in some subgroups. However, these intervals relate to the secondary aim of this study. Additionally, a sensitivity analysis showed very little change to estimates when problematic variables were removed, indicating that results concerning associations were robust (see Supplementary Table S7).

The diagnostic accuracy of serological testing is variable across different antigens and testing platforms, and sensitivity and specificity of the tests used are <100%. This study did not make any adjustment for assay performance. However, standard methods were followed, and results are comparable to other high-volume seroprevalence studies. A subset of samples were tested at VIDRL, Australia's measles reference laboratory, to check concordance of measles antibody concentrations (see Supplementary Figure S6). While reference testing appeared to returned slightly lower measles IgG antibody concentrations when compared index testing, the degree of concordance in this verification exercise was considered acceptable, while acknowledging that samples had undergone at least one additional freeze–thaw cycle and had been transported cross-border between tests (potentially affecting sample quality). Easier access and more reliable transport to a reference testing centre would have allowed further investigation into this potential affect. Nevertheless, available data suggest that, if present, any bias could be towards overestimation of measles IgG seroprevalence in Timor-Leste (i.e. that the true seroprevalences are lower than reported).

To use serosurveys to assess vaccine uptake, an assumption that seronegative individuals are unvaccinated (and seropositive individuals are either vaccinated or previously infected) must be made. However, the likelihood of seroconversion after vaccination and the duration of seropositivity depends on various vaccine-related factors (number of doses and their quality at the time of administration) and host-related factors (age at vaccination and whether malnutrition or other immunosuppression is present).^22^^,^^36^, ^37^, ^38^ Without adjusting for these effects, direct translation from seroprevalence may result in underestimation of rates of vaccine uptake. This study did not collect individual data on routine vaccinations, apart from SARS-CoV-2 vaccination, because fieldwork pilots indicated that information would be difficult and time-consuming to locate and verify.

A further assumption that seropositive individuals are all protected against infection upon subsequent exposure (and seronegative individuals are all susceptible) must be made. For measles, WHO and other groups recommend using 120 IU/L as the most appropriate quantitative serological cut-off.^2^^,^^15^^,^^16^ However, evidence that this correlate-of-protection translates to immunity is limited.^14^^,^^39^ Alternative approaches to setting serological cutoffs include mixture modelling and testing groups of known susceptible (and/or known immune) individuals, but these methods would have limited the comparability of data from this study to other serosurveys.^40^

### Conclusion

This study determined seroprevalence of four priority VPDs in relevant age-strata of the Timor-Leste general population, providing a detailed cross sectional profile reflecting patterns of vaccine and infection-derived immunity. It identified significant national immunity gaps against measles and hepatitis B in children, which informs the need for interventions to improve uptake of routine vaccines, catch up vaccination and targeted SIAs. Compared to measles IgG, rubella IgG seroprevalence was high, indicating likely ongoing transmission. Ongoing case-based surveillance for VPDs based on the Timor-Leste Integrated Disease Surveillance and Response guideline is essential.^41^

## Contributors

PA, MYT, NM, SLS, ADKD, NS, CF, FNM, NSSF, KM, JY and JRF conceived and designed the study. MYT, VS, SA and LA coordinated fieldwork, enrolled participants and collected epidemiological data and samples. PA, NG, TO, ES, LA, SN, VS and KP lead the serological analysis of samples. PA, VS, MD, SLS, TWY and ADKD were responsible for statistical analysis. PA drafted the manuscript, with all authors having significant contribution to revisions, finalisations for submission and decision to submit.

## Data sharing statement

Study data are owned by Instituto Nacional de Saúde Pública, Timor-Leste Ministry of Health. Individual participant data that underlie the results reported in this article, after de-identification (text, tables, figures, and appendices) and will be made available upon reasonable request beginning 9 months and ending 36 months following article publication by investigators whose proposed use of the data has been approved by an independent review committee (“learned intermediary”) identified for this purpose, for individual participant data meta-analysis. Proposals should be directed to paul.arkell@menzies.edu.au; to gain access, data requestors will need to sign a data access agreement.

## Editor note

The Lancet Group takes a neutral position with respect to territorial claims in published maps and institutional affiliations.

## Declaration of interests

Authors do not have any commercial or other associations that might pose conflicts of interest.
