# Supplementary material for: A population-representative serosurvey estimating vaccine-induced immunity against measles, rubella, hepatitis B and severe acute respiratory syndrome coronavirus 2 in Timor-Leste
Source: Lancet Reg Health Southeast Asia. 2025 Feb 25;34:100525. doi: 10.1016/j.lansea.2024.100525 (PMC11905827; doi:10.1016/j.lansea.2024.100525)
Supplement: Supplementary Material [file mmc1.pdf]

A population-representative serosurvey estimating vaccine-induced immunity against measles, rubella, hepatitis B and severe acute respiratory syndrome coronavirus 2 in Timor-Leste

Supplementary Material

Table of Contents

Supplementary Table 1 3

Supplementary Figure 1 4

Supplementary Figure 2 5

Supplementary Figure 3 6

Supplementary Figure 4 7

Supplementary Figure 5 8

Supplementary Figure 6 9

Supplementary Table 1: Summary of participating households (N=1908) and comparison to non-participating households (N=705)

Household-level characteristic	**Participating households (n=1908)**	**Non-participating households (n=705)**	**TOTAL (n=2613)**
Municipality (%)			
- Aileu	75 (3.9)	40 (5.7)	115 (4.4)
- Ainaro	105 (5.5)	35 (5.0)	140 (5.4)
- Baucau	222 (11.6)	32 (4.5)	254 (9.7)
- Bobonaro	141 (7.4)	44 (6.2)	185 (7.1)
- Covalima	124 (6.5)	14 (2.0)	138 (5.3)
- Dili	543 (28.5)	127 (18.0)	670 (25.6)
- Ermera	120 (6.3)	160 (22.7)	280 (10.7)
- Lautem	102 (5.3)	39 (5.5)	141 (5.4)
- Liquiçá	84 (4.4)	78 (11.1)	162 (6.2)
- Manatuto	85 (4.5)	11 (1.6)	96 (3.7)
- Manufahi	60 (3.1)	54 (7.7)	114 (4.4)
- Oecusse	128 (6.7)	26 (3.7)	154 (5.9)
- Viqueque	119 (6.2)	45 (6.4)	164 (6.3)
Mean distance by road to CHC (SD)	4.796 (1.7-10.902)	6.4 (2.2-12.793)	5.028 (1.787-11.333)
Assignment during 2015 National Census (%)			
- Urban	590 (30.9)	137 (19.4)	727 (27.8)
- Rural	1318 (69.1)	568 (80.6)	1886 (72.2)
**TOTAL**	**1908**	**705**	**2613**

Abbreviations: CHC = community health centre, IQR = interquartile range

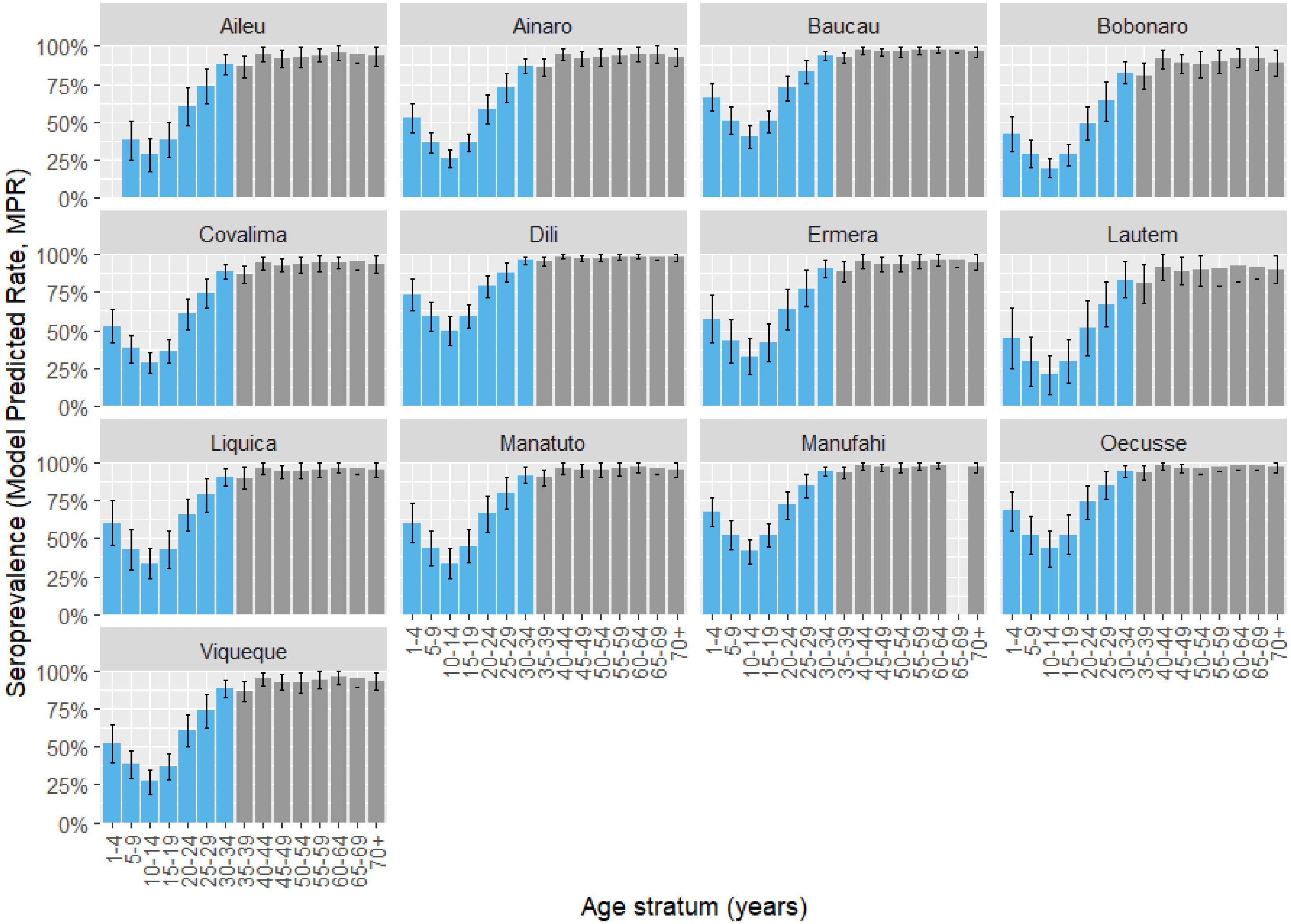

Supplementary Figure 1: Model-predicted measles IgG seroprevalence estimates for municipalities in Timor-Leste. Vaccine-eligible age strata are shown in blue.

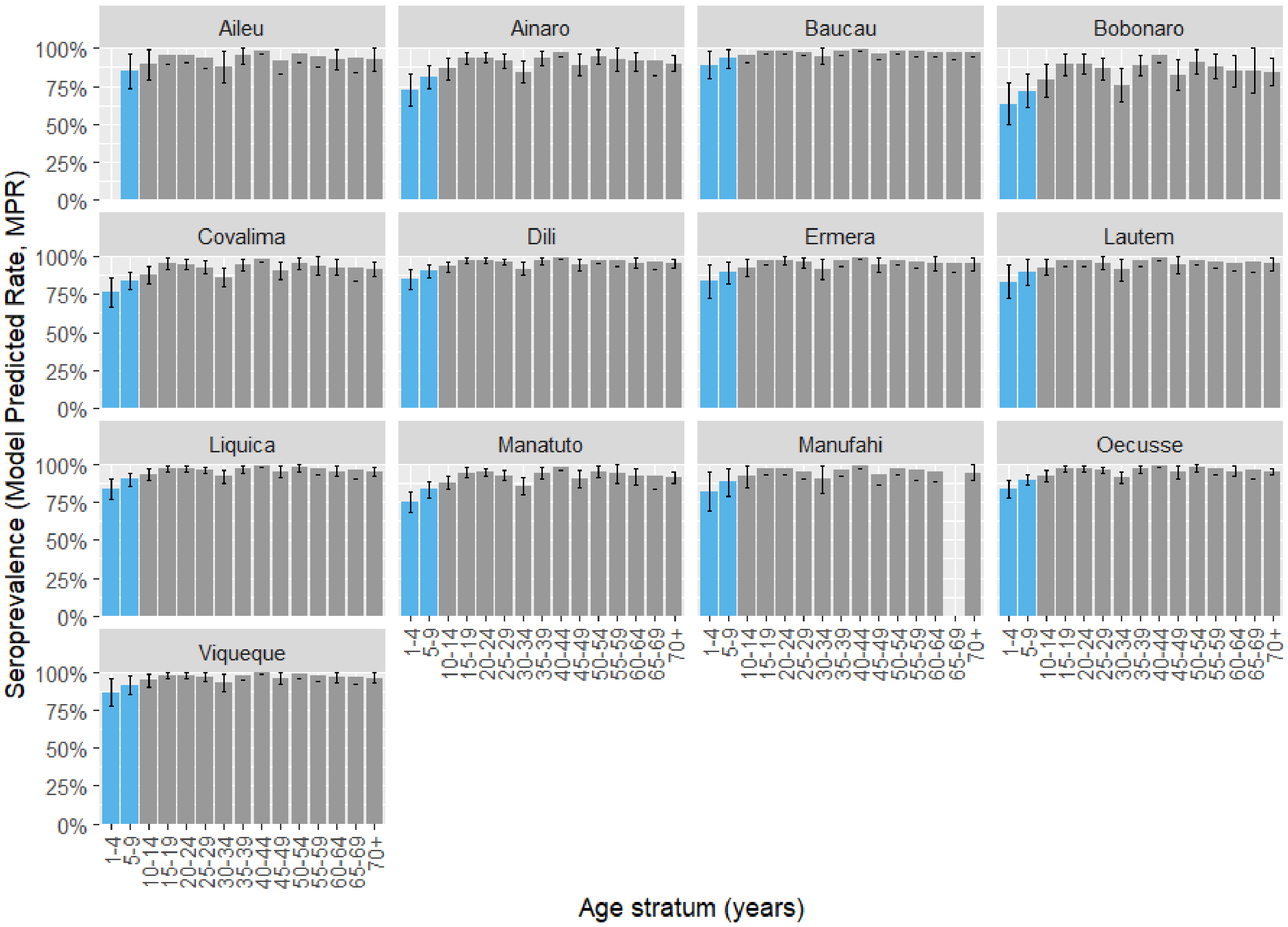

Supplementary Figure 2: Model-predicted rubella IgG seroprevalence estimates for municipalities in Timor-Leste. Vaccine-eligible age strata are shown in blue.

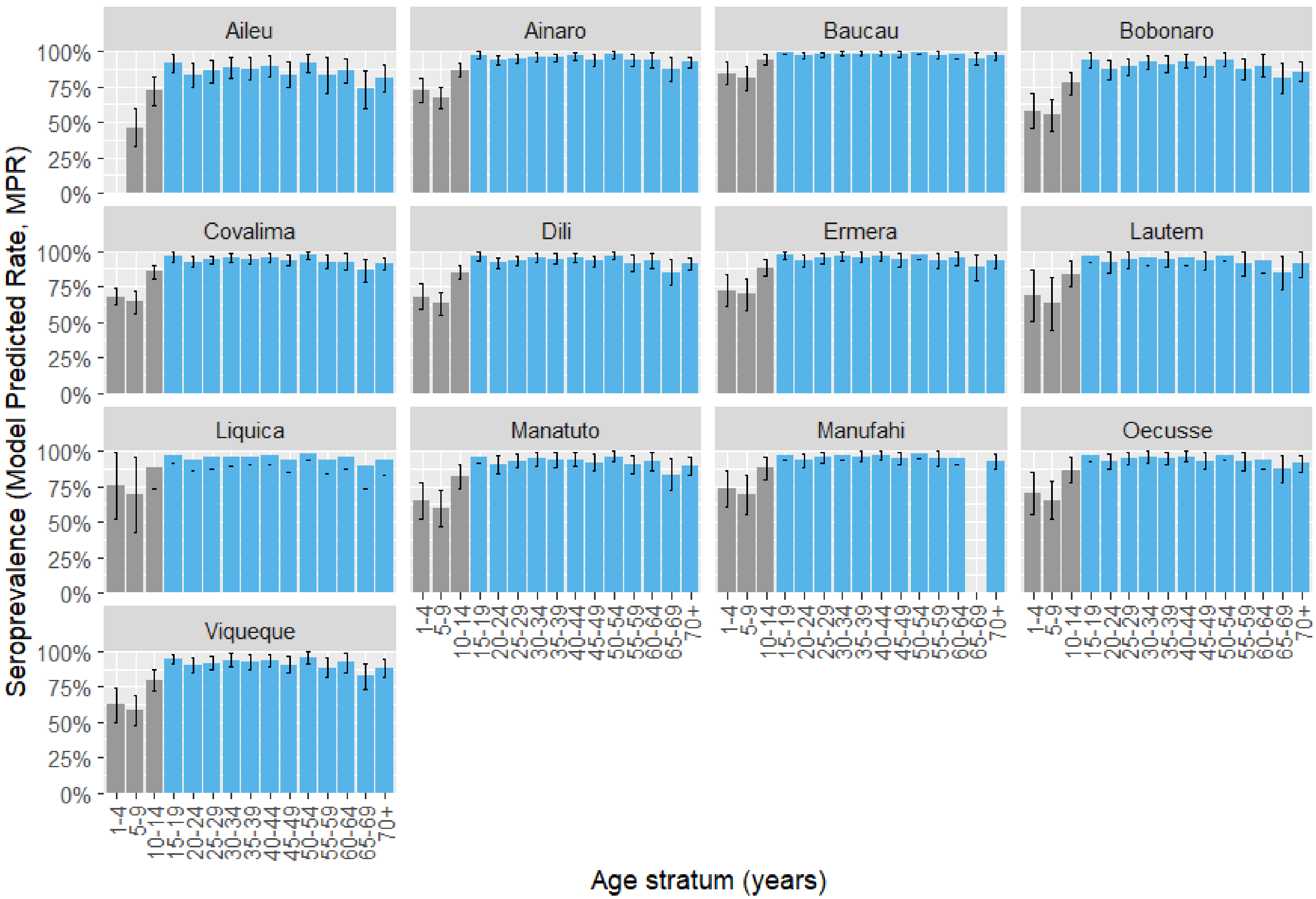

Supplementary Figure 3: Model-predicted severe acute respiratory syndrome coronavirus 2 (SARS-CoV-2) anti-spike IgG seroprevalence estimates for municipalities in Timor-Leste. Vaccine-eligible age strata are shown in blue.

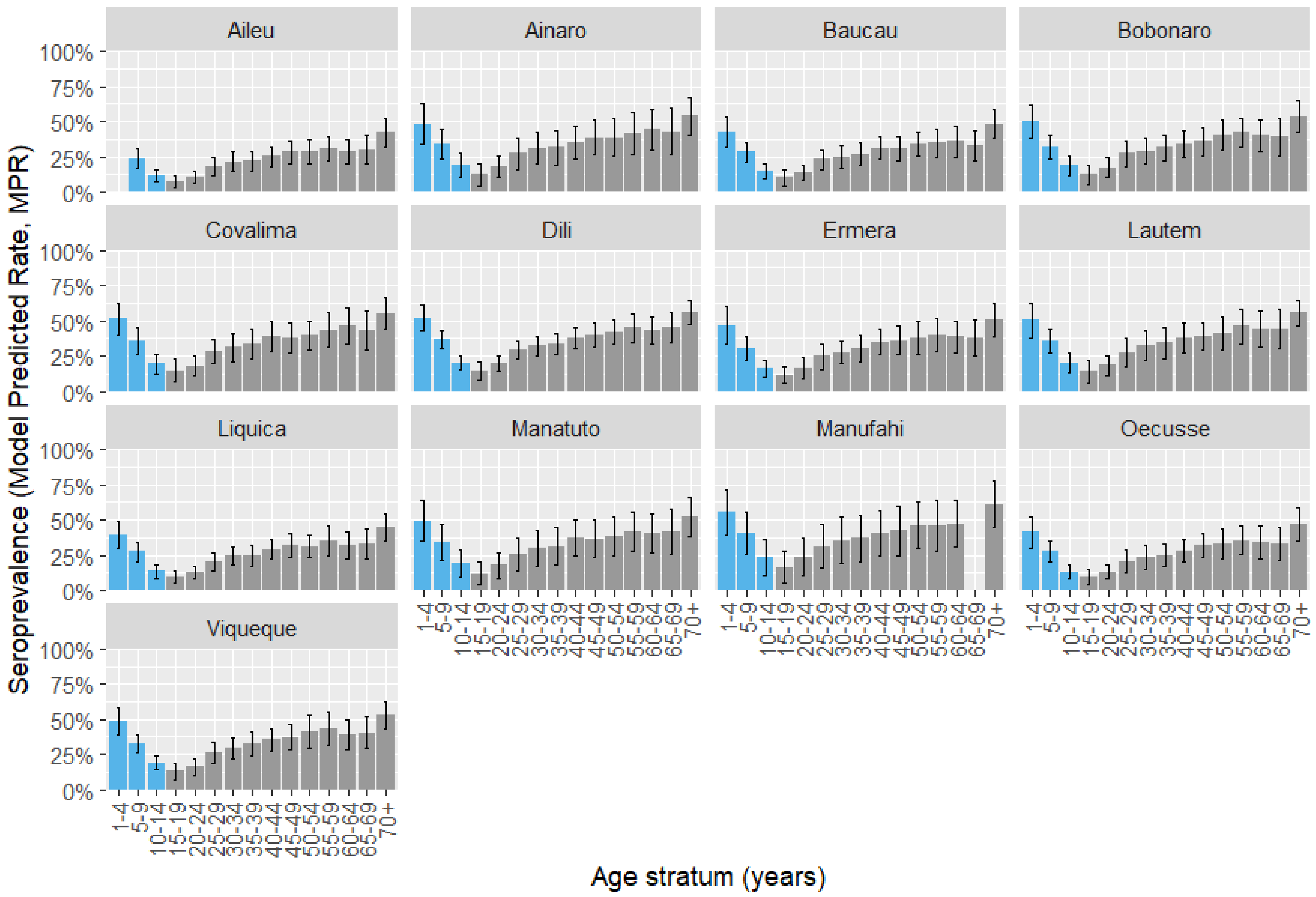

Supplementary Figure 4: Model-predicted hepatitis B surface antibody (HBsAb) seroprevalence estimates for municipalities in Timor-Leste. Vaccine-eligible age strata are shown in blue.

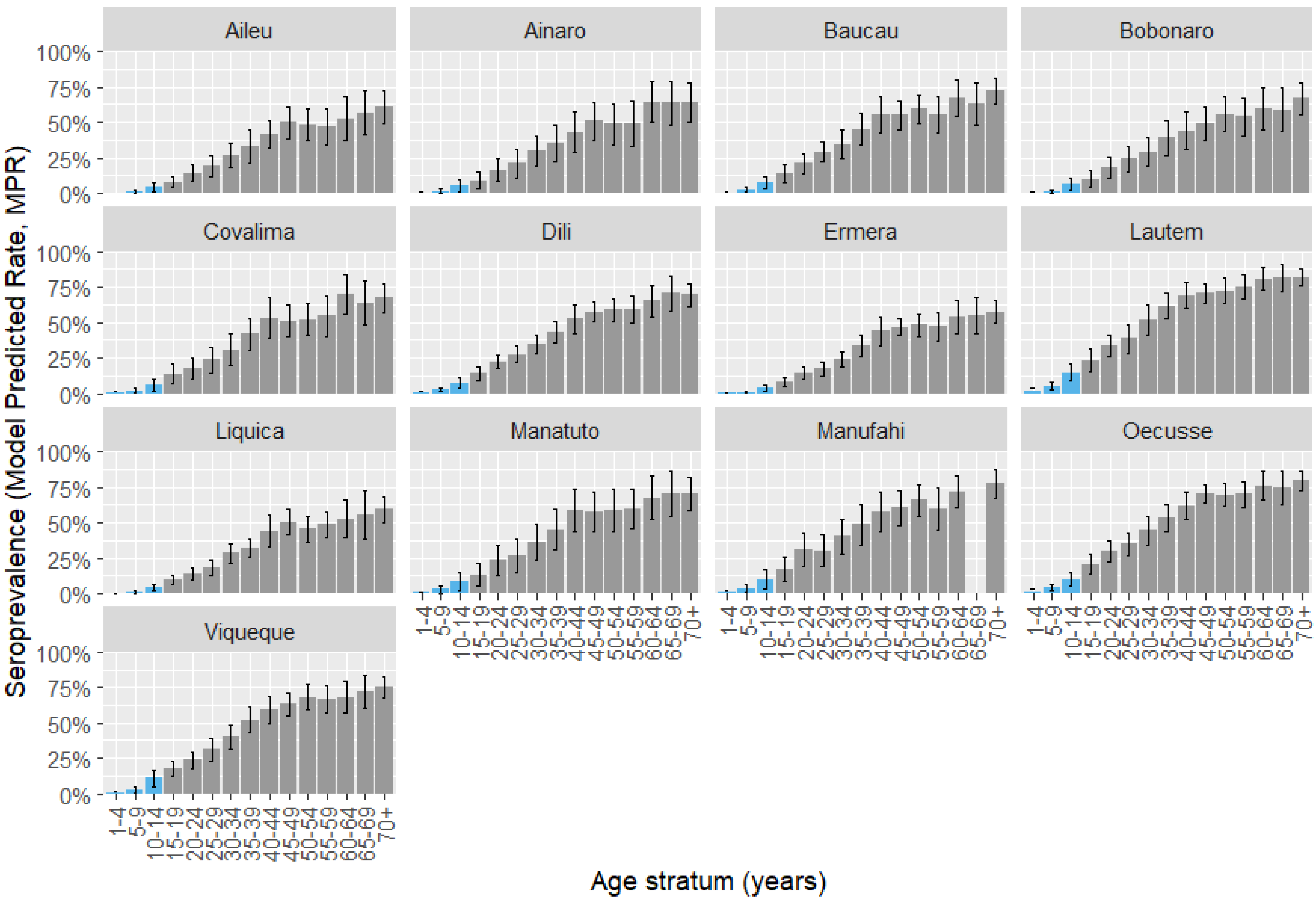

Supplementary Figure 5: Model-predicted hepatitis B core antibody (HBcAb) seroprevalence estimates for municipalities in Timor-Leste. Vaccine-eligible age strata are shown in blue.

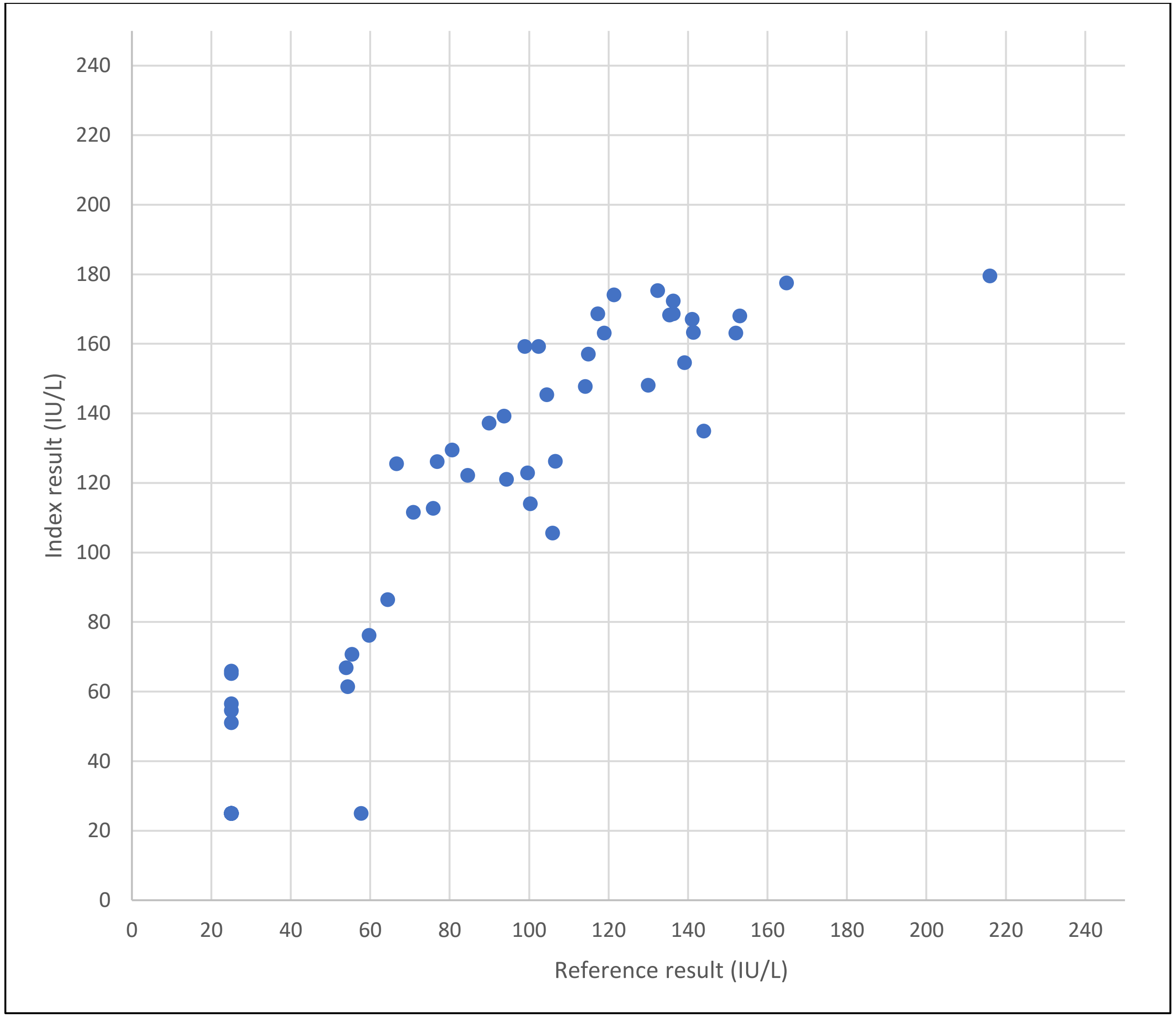

Supplementary Figure 6: Scatter plot showing verification of quantitative measles IgG results from Laboratório Nacional da Saúde, Dili (LNS, index result, y-axis) by retesting them at the Victorian Infectious Diseases Reference Laboratory (VIDRL, reference result, x-axis). A subset of 16 samples which were determined negative and 30 samples determined low positive at LNS were included.
