# Supplementary material for: A population-representative serosurvey estimating vaccine-induced immunity against measles, rubella, hepatitis B and severe acute respiratory syndrome coronavirus 2 in Timor-Leste
Source: Lancet Reg Health Southeast Asia. 2025 Feb 25;34:100525. doi: 10.1016/j.lansea.2024.100525 (PMC11905827; doi:10.1016/j.lansea.2024.100525)
Supplement: Supplementary Tables [file mmc2.docx]

Supplementary Table 2: Associations of individual and household characteristics with Measles IgG seropositivity. Data are replicated from main text Table 3, with addition of event size statistics.

|  | **Measles IgG (N=2891)** | | | | |
| --- | --- | --- | --- | --- | --- |
|  | **n** | **Detected** | **OR** | **(95%CI)** | **p value** |
| **Fixed effects**  Age stratum  - 1-4 years  - 5-9 years  - 10-14 years  - 15-19 years  - 20-24 years  - 25-29 years  - 30-34 years  - 35-39 years  - 40-44 years  - 45-49 years  - 50-54 years  - 55-59 years  - 60-64 years  - 65-69 years  - 70+ years | 271  545  494  390  422  378  391  -  -  -  -  -  -  -  - | 145  228  154  188  292  307  344  -  -  -  -  -  -  -  - | ref  0.34  0.17  0.33  1.35  3.69  16.44  -  -  -  -  -  -  -  - | ref  (0.16-0.73)  (0.08-0.38)  (0.16-0.72)  (0.49-3.73)  (1.28-10.70)  (6.91-39.11)  -  -  -  -  -  -  -  - | ref  0.006  <0.001  0.005  0.564  0.017  <0.001  -  -  -  -  -  -  -  - |
| Gender  -Female  - Male | 1690  1201 | 1043  615 | ref  0.65 | ref  (0.48-0.87) | ref  0.005 |
| HH occupancy | - | - | 1.01 | (0.95-1.08) | 0.701 |
| Recent fever | - | - | - | - | - |
| SARS-CoV-2 vaccination | - | - | - | - | - |
| Day of outbreak | - | - | - | - | - |
| Municipality  - Dili  - Aileu  - Ainaro  - Baucau  - Bobonaro  - Covalima  - Ermera  - Lautem  - Liquiçá  - Manatuto  - Manufahi  - Oecusse  - Viqueque | 844  108  191  242  218  205  199  159  123  149  111  155  187 | 347  61  88  151  91  108  110  79  75  84  61  96  105 | ref  0.25  0.17  0.13  0.05  0.19  0.30  0.18  0.39  0.24  0.31  0.53  0.21 | ref  (0.08 -0.82)  (0.06-0.43)  (0.15-0.96)  (0.02-0.18)  (0.06-0.59)  (0.10-0.84)  (0.05-0.68)  (0.14-1.10)  (0.06-0.91)  (0.12-0.82)  (0.19-1.52)  (0.08-0.56) | ref  0.023  <0.001  0.040  <0.001  0.004  0.023  0.012  0.075  0.036  0.019  0.235  0.002 |
| Log (dist to CHC) | - | - | 1.33 | (0.94-1.88) | 0.109 |
| Log (EA pop density) | - | - | 0.98 | (0.83-1.15) | 0.787 |
| Household location  - Rural  - Urban | 2016  875 | 1099  559 | ref  1.25 | ref  (0.55-2.86) | ref  0.588 |
| **Random effects**  Enumeration area | - | - | 0.79 | (0.44-1.44) | - |
| Household | - | - | 3.35 | (2.33-4.82) | - |

Supplementary Table 3: Associations of individual and household characteristics with Rubella IgG seropositivity. Data are replicated from main text Table 3, with addition of event size statistics.

|  | **Rubella IgG (N=801)** | | | | |
| --- | --- | --- | --- | --- | --- |
|  | **n** | **Detected** | **OR** | **(95%CI)** | **p value** |
| **Fixed effects**  Age stratum  - 1-4 years  - 5-9 years  - 10-14 years  - 15-19 years  - 20-24 years  - 25-29 years  - 30-34 years  - 35-39 years  - 40-44 years  - 45-49 years  - 50-54 years  - 55-59 years  - 60-64 years  - 65-69 years  - 70+ years | 260  541  -  -  -  -  -  -  -  -  -  -  -  -  - | 204  471  -  -  -  -  -  -  -  -  -  -  -  -  - | ref  2.10  -  -  -  -  -  -  -  -  -  -  -  -  - | ref  (0.79-5.55)  -  -  -  -  -  -  -  -  -  -  -  -  - | ref  0.135  -  -  -  -  -  -  -  -  -  -  -  -  - |
| Gender  -Female  - Male | 405  396 | 346  329 | ref  1.20 | ref  (0.43-2.84) | ref  0.843 |
| HH occupancy | - | - | 0.87 | (0.67-0.13) | 0.287 |
| Recent fever | - | - | - | - | - |
| SARS-CoV-2 vaccination | - | - | - | - | - |
| Day of outbreak | - | - | - | - | - |
| Municipality  - Dili  - Aileu  - Ainaro  - Baucau  - Bobonaro  - Covalima  - Ermera  - Lautem  - Liquiçá  - Manatuto  - Manufahi  - Oecusse  - Viqueque | 200  19  59  67  53  71  46  64  31  48  41  53  49 | 177  18  38  63  36  57  41  58  27  40  33  44  43 | ref  5.79  0.01  7.23  0.03  0.00  0.50  0.13  1.50  0.26  0.12  0.09  0.61 | ref  (0.01- 7104.57)  (0.00-0.12)  (0.11-474.37)  (0.00-2.79)  (0.00-0.62)  (0.01-23.30)  (0.00-60.67)  (0.12-179.78)  (0.01-10.28)  (0.00-23.65)  (0.01-1.20)  (0.01-26.90) | ref  0.625  0.001  0.350  0.127  0.023  0.720  0.507  0.868  0.469  0.431  0.068  0.795 |
| Log (dist to CHC) | - | - | 1.30 | (0.35-4.85) | 0.695 |
| Log (EA pop density) | - | - | 1.74 | (0.75-4.03) | 0.196 |
| Household location  - Rural  - Urban | 595  206 | 500  175 | ref  0.04 | ref  (0.00-0.39) | ref  0.007 |
| **Random effects**  Enumeration area | - | - | 13.04 | (5.85 -29.08) | - |
| Household | - | - | 9.149 | (4.07-20.57) | - |

Supplementary Table 4: Associations of individual and household characteristics with SARS-CoV-2 IgG seropositivity. Data are replicated from main text Table 3, with addition of event size statistics.

|  | **ScV2 anti-S IgG (N=3416)** | | | | |
| --- | --- | --- | --- | --- | --- |
|  | **n** | **Detected** | **OR** | **(95%CI)** | **p value** |
| **Fixed effects**  Age stratum  - 1-4 years  - 5-9 years  - 10-14 years  - 15-19 years  - 20-24 years  - 25-29 years  - 30-34 years  - 35-39 years  - 40-44 years  - 45-49 years  - 50-54 years  - 55-59 years  - 60-64 years  - 65-69 years  - 70+ years | -  -  -  387  417  375  390  336  252  292  245  153  179  112  278 | -  -  -  370  398  352  376  311  241  275  236  144  167  95  255 | -  -  -  ref  0.14  0.11  0.23  0.15  0.24  0.13  1.19  0.07  0.18  0.01  0.04 | -  -  -  ref  (0.03-0.68)  (0.02-0.70)  (0.03-1.67)  (0.02-1.05)  (0.04-1.30)  (0.02-0.76)  (0.11-13.51)  (0.01-0.52)  (0.02-1.67)  (0.00-0.06)  (0.01-0.27) | -  -  -  ref  0.016  0.020  0.146  0.056  0.096  0.024  0.887  0.011  0.129  <0.001  0.001 |
| Gender  -Female  - Male | 2138  1278 | 2032  1188 | ref  0.60 | ref  (0.31-1.18) | ref  0.136 |
| HH occupancy | - | - | 0.95 | (0.77-1.17) | 0.628 |
| Recent fever | 254 | 242 | 1.19 | (0.27-5.18) | 0.814 |
| SARS-CoV-2 vaccination | 2729 | 2644 | 55.85 | (14.5-214.54) | <0.001 |
| Day of outbreak | - | - | 1.01 | (1.00-1.03) | 0.079 |
| Municipality  - Dili  - Aileu  - Ainaro  - Baucau  - Bobonaro  - Covalima  - Ermera  - Lautem  - Liquiçá  - Manatuto  - Manufahi  - Oecusse  - Viqueque | 861  148  188  335  286  247  248  206  177  155  118  221  226 | 797  130  179  329  259  241  235  194  162  148  115  213  218 | ref  0.30  3.17  18.95  2.84  1.61  1.49  2.08  8.83  3.12  15.59  0.87  2.25 | ref  (0.03-3.11)  (0.05-202.86)  (0.11-3377.28)  (0.13-63.26)  (0.06-43.78)  (0.05-49.11)  (0.07-58.67)  (0.35-220.26)  (0.05-218.12)  (0.05- 4894.98)  (0.01-156.82)  (0.07-70.85) | ref  0.307  0.582  0.262  0.507  0.776  0.820  0.663  0.182  0.596  0.345  0.956  0.642 |
| Log (dist to CHC) | - | - | 1.93 | (0.98-3.79) | 0.057 |
| Log (EA pop density) | - | - | 0.70 | (0.37-1.30) | 0.255 |
| Household location  - Rural  - Urban | 2497  919 | 2357  863 | ref  99.67 | ref  (4.25-2338.61) | ref  0.005 |
| **Random effects**  Enumeration area | - | - | 10.15 | (4.93-20.92) | - |
| Household | - | - | 19.13 | (8.00-45.76) | - |

Supplementary Table 5: Associations of individual and household characteristics with hepatitis B surface antibody (HBsAb) seropositivity. Data are replicated from main text Table 3, with addition of event size statistics.

|  | **HBsAb (N=1290)** | | | | |
| --- | --- | --- | --- | --- | --- |
|  | **n** | **Detected** | **OR** | **(95%CI)** | **p value** |
| **Fixed effects**  Age stratum  - 1-4 years  - 5-9 years  - 10-14 years  - 15-19 years  - 20-24 years  - 25-29 years  - 30-34 years  - 35-39 years  - 40-44 years  - 45-49 years  - 50-54 years  - 55-59 years  - 60-64 years  - 65-69 years  - 70+ years | 260  540  490  -  -  -  -  -  -  -  -  -  -  -  - | 124  182  88  -  -  -  -  -  -  -  -  -  -  -  - | ref  0.34  0.05  -  -  -  -  -  -  -  -  -  -  -  - | ref  (0.16-0.74)  (0.02-0.13)  -  -  -  -  -  -  -  -  -  -  -  - | ref  0.007  <0.001  -  -  -  -  -  -  -  -  -  -  -  - |
| Gender  -Female  - Male | 648  642 | 186  208 | ref  1.37 | ref  (0.84-2.25) | ref  0.203 |
| HH occupancy | - | - | 1.06 | (0.91-1.22) | 0.462 |
| Recent fever | - | - | - | - | - |
| SARS-CoV-2 vaccination | - | - | - | - | - |
| Day of outbreak | - | - | - | - | - |
| Municipality  - Dili  - Aileu  - Ainaro  - Baucau  - Bobonaro  - Covalima  - Ermera  - Lautem  - Liquiçá  - Manatuto  - Manufahi  - Oecusse  - Viqueque | 332  33  97  106  92  107  79  107  48  80  58  77  74 | 108  11  31  25  22  38  30  25  20  26  20  17  21 | ref  2.27  0.74  0.44  2.45  2.00  3.64  0.46  1.15  1.95  1.25  0.50  0.85 | ref  (0.15-34.23)  (0.15-3.74)  (0.06-3.46)  (0.19-32.07)  (0.24-16.64)  (0.54-24.51)  (0.05-3.89)  (0.05-26.35)  (0.32-11.93)  (0.22-7.27)  (0.11-2.32)  (0.09-8.03) | ref  0.550  0.717  0.427  0.490  0.516  0.182  0.470  0.929  0.466  0.802  0.376  0.884 |
| Log (dist to CHC) | - | - | 1.17 | (0.85-1.63) | 0.335 |
| Log (EA pop density) | - | - | 1.36 | (0.86-2.17) | 0.187 |
| Household location  - Rural  - Urban | 939  351 | 293  101 | ref  0.23 | ref  (0.49-1.08) | ref  0.062 |
| **Random effects**  Enumeration area | - |  | 3.55 | (1.69-7.46) | - |
| Household | - |  | 6.19 | (3.68-10.40) | - |

Supplementary Table 6: Associations of individual and household characteristics with hepatitis B core antibody (HBcAb) seropositivity. Data are replicated from main text Table 3, with addition of event size statistics.

|  | **HBcAb (N=1290)** | | | | |
| --- | --- | --- | --- | --- | --- |
|  | **n** | **Detected** | **OR** | **(95%CI)** | **p value** |
| **Fixed effects**  Age stratum  - 1-4 years  - 5-9 years  - 10-14 years  - 15-19 years  - 20-24 years  - 25-29 years  - 30-34 years  - 35-39 years  - 40-44 years  - 45-49 years  - 50-54 years  - 55-59 years  - 60-64 years  - 65-69 years  - 70+ years | 260  540  490  -  -  -  -  -  -  -  -  -  -  -  - | 2  13  24  -  -  -  -  -  -  -  -  -  -  -  - | ref  13.14  97.25  -  -  -  -  -  -  -  -  -  -  -  - | ref  (0.26-6685.54)  (0.04-217359.00)  -  -  -  -  -  -  -  -  -  -  -  - | ref  0.414  0.241  -  -  -  -  -  -  -  -  -  -  -  - |
| Gender  -Female  - Male | 648  642 | 12  27 | ref  5.67 | ref  (0.73-44.23) | ref  0.996 |
| HH occupancy | - | - | 1.09 | (0.56-2.151) | 0.797 |
| Recent fever | - | - | - | - | - |
| SARS-CoV-2 vaccination | - | - | - | - | - |
| Day of outbreak | - | - | - | - | - |
| Municipality  - Dili  - Aileu  - Ainaro  - Baucau  - Bobonaro  - Covalima  - Ermera  - Lautem  - Liquiçá  - Manatuto  - Manufahi  - Oecusse  - Viqueque | 332  33  97  106  92  107  79  107  48  80  58  77  74 | 8  1  2  3  1  3  2  4  1  5  3  2  4 | ref  0.86  0.56  1.94  0.05  0.94  3.32  25.32  0.13  13.80  6.13  3.56  1.66 | ref  (0.00-2.73x10^23^)  (0.00-2.28x10^8^)  (0.00-4.97x10^6^)  (0.00-1145.47)  (0.00-2.82x10^14^)  (0.00-3.66x10^5^)  (0.00-1.14x10^13^)  (0.00-3564.29)  (0.00-7.77x10^8^)  (0.00-1.47x10^7^)  (0.00-8.69x10^7^)  (0.00-1.95x10^16^) | ref  0.996  0.953  0.929  0.553  0.997  0.838  0.811  0.694  0.771  0.807  0.882  0.978 |
| Log (dist to CHC) | - | - | 1.89 | (0.13-27.55) | 0.797 |
| Log (EA pop density) | - | - | 0.47 | (0.02-12.51) | 0.645 |
| Household location  - Rural  - Urban | 939  351 | 31  8 | ref  9.46 | ref  (0.02-4960.03) | ref  0.478 |
| **Random effects**  Enumeration area | - | - | 20.97 | (2.28-193.09) | - |
| Household | - | - | 17.06 | (0.20-1428.31) | - |

**Supplementary Table 7**: Sensitivity analysis of SARS-CoV-2 IgG modelling showing very little difference in point and precision estimates when variables with wide confidence intervals are removed.

|  | **Original** | | | **Variables with wide CIs removed** | | |
| --- | --- | --- | --- | --- | --- | --- |
|  | **OR** | **95%CI** | **p-value** | **OR** | **95%CI** | **p-value** |
| **Fixed effects** |  |  |  |  |  |  |
| **Age stratum** |  |  |  |  |  |  |
| **- 1-4 years** | - | - | - | - | - | - |
| **- 5-9 years** | - | - | - | - | - | - |
| **- 10-14 years** | - | - | - | - | - | - |
| **- 15-19 years** | ref | ref | ref | ref | ref | ref |
| **- 20-24 years** | 0.14 | (0.03-0.68) | 0.016 | 0.13 | (0.03 - 0.68) | 0.016 |
| **- 25-29 years** | 0.11 | (0.02-0.70) | 0.020 | 0.11 | (0.02 - 0.72) | 0.022 |
| **- 30-34 years** | 0.23 | (0.03-1.67) | 0.146 | 0.23 | (0.03 - 1.75) | 0.154 |
| **- 35-39 years** | 0.15 | (0.02-1.05) | 0.056 | 0.15 | (0.02 - 1.10) | 0.062 |
| **- 40-44 years** | 0.24 | (0.04-1.30) | 0.096 | 0.23 | (0.04 - 1.40) | 0.109 |
| **- 45-49 years** | 0.13 | (0.02-0.76) | 0.024 | 0.13 | (0.02 - 0.79) | 0.028 |
| **- 50-54 years** | 1.19 | (0.11-13.51) | 0.887 | 1.19 | (0.10 - 14.52) | 0.889 |
| **- 55-59 years** | 0.07 | (0.01-0.52) | 0.011 | 0.07 | (0.01 - 0.55) | 0.013 |
| **- 60-64 years** | 0.18 | (0.02-1.67) | 0.129 | 0.18 | (0.02 - 1.76) | 0.139 |
| **- 65-69 years** | 0.01 | (0.00-0.06) | <0.001 | 0.01 | (0.00 - 0.06) | <0.001 |
| **- 70+ years** | 0.04 | (0.01-0.27) | 0.001 | 0.04 | (0.01 - 0.28) | 0.001 |
| **Gender** |  |  |  |  |  |  |
| **-Female** | ref | ref | ref | ref | ref | ref |
| **- Male** | 0.6 | (0.31-1.18) | 0.136 | 0.59 | (0.30 - 1.18) | 0.135 |
| **HH occupancy** | 0.95 | (0.77-1.17) | 0.628 | 0.95 | (0.77 - 1.19) | 0.67 |
| **Recent fever** | 1.19 | (0.27-5.18) | 0.814 | 1.17 | (0.27 - 5.13) | 0.836 |
| **SARS-CoV-2 vac.** | 55.85 | (14.5-214.54) | <0.001 | 57.39 | (13.79 - 238.86) | <0.001 |
| **Day of outbreak** | 1.01 | (1.00-1.03) | 0.079 | 1.01 | (1.00 - 1.02) | 0.337 |
| **Municipality** |  |  |  |  |  |  |
| **- Dili** | ref | ref | ref | - | - | - |
| **- Aileu** | 0.30 | (0.03-3.11) | 0.307 | - | - | - |
| **- Ainaro** | 3.17 | (0.05-202.86) | 0.582 | - | - | - |
| **- Baucau** | 18.95 | (0.11-3377.28) | 0.262 | - | - | - |
| **- Bobonaro** | 2.84 | (0.13-63.26) | 0.507 | - | - | - |
| **- Covalima** | 1.61 | (0.06-43.78) | 0.776 | - | - | - |
| **- Ermera** | 1.49 | (0.05-49.11) | 0.82 | - | - | - |
| **- Lautem** | 2.08 | (0.07-58.67) | 0.663 | - | - | - |
| **- Liquiçá** | 8.83 | (0.35-220.26) | 0.182 | - | - | - |
| **- Manatuto** | 3.12 | (0.05-218.12) | 0.596 | - | - | - |
| **- Manufahi** | 15.59 | (0.05- 4894.98) | 0.345 | - | - | - |
| **- Oecusse** | 0.87 | (0.01-156.82) | 0.956 | - | - | - |
| **- Viqueque** | 0.70 | (0.07-70.85) | 0.642 | - | - | - |
| **Log (dist to CHC)** | 1.93 | (0.98-3.79) | 0.057 | - | - | - |
| **Log (EA pop dens.)** | 0.7 | (0.37-1.30) | 0.255 | - | - | - |
| **Household location** |  |  |  |  |  |  |
| **- Rural** | ref | ref | ref | - | - | - |
| **- Urban** | 99.67 | (4.25-2338.61) | 0.005 | - | - | - |
